# Chilling stress drives organ-specific transcriptional cascades and dampens diurnal oscillation in tomato

**DOI:** 10.1093/hr/uhad137

**Published:** 2023-07-11

**Authors:** Tina Agarwal, Xiaojin Wang, Frederick Mildenhall, Iskander M Ibrahim, Sujith Puthiyaveetil, Kranthi Varala

**Affiliations:** Department of Horticulture and Landscape Architecture, Purdue University, West Lafayette, IN 47907, USA; Purdue Center for Plant Biology, Purdue University, West Lafayette, IN 47907, USA; Department of Horticulture and Landscape Architecture, Purdue University, West Lafayette, IN 47907, USA; Purdue Center for Plant Biology, Purdue University, West Lafayette, IN 47907, USA; Department of Horticulture and Landscape Architecture, Purdue University, West Lafayette, IN 47907, USA; Purdue Center for Plant Biology, Purdue University, West Lafayette, IN 47907, USA; Department of Horticulture and Landscape Architecture, Purdue University, West Lafayette, IN 47907, USA; Department of Biochemistry, Purdue University, West Lafayette, IN 47907, USA; Department of Horticulture and Landscape Architecture, Purdue University, West Lafayette, IN 47907, USA; Department of Biochemistry, Purdue University, West Lafayette, IN 47907, USA; Department of Horticulture and Landscape Architecture, Purdue University, West Lafayette, IN 47907, USA; Purdue Center for Plant Biology, Purdue University, West Lafayette, IN 47907, USA

## Abstract

Improving chilling tolerance in cold-sensitive crops, e.g. tomato, requires knowledge of the early molecular response to low temperature in these under-studied species. To elucidate early responding processes and regulators, we captured the transcriptional response at 30 minutes and 3 hours in the shoots and at 3 hours in the roots of tomato post-chilling from 24°C to 4°C. We used a pre-treatment control and a concurrent ambient temperature control to reveal that majority of the differential expression between cold and ambient conditions is due to severely compressed oscillation of a large set of diurnally regulated genes in both the shoots and roots. This compression happens within 30 minutes of chilling, lasts for the duration of cold treatment, and is relieved within 3 hours of return to ambient temperatures. Our study also shows that the canonical ICE1/CAMTA-to-CBF cold response pathway is active in the shoots, but not in the roots. Chilling stress induces synthesis of known cryoprotectants (trehalose and polyamines), in a CBF-independent manner, and induction of multiple genes encoding proteins of photosystems I and II. This study provides nuanced insights into the organ-specific response in a chilling sensitive plant, as well as the genes influenced by an interaction of chilling response and the circadian clock.

## Introduction

Plant response to low temperature occurs via transcriptional reprogramming and translational regulation of genes, and leads to metabolic adjustments that protect the plant from cold exposure [1, 2]. It includes activation of signaling pathways, hormone transduction pathways, lipid metabolism etc., and thus involves a complex gene regulatory network (GRN) [1]. Much of the molecular knowledge of cold response was learnt from the temperate model plant *Arabidopsis thaliana* [3]. Through these studies, the CRT binding factor (CBF) family of transcription factors were identified as primary regulators of plant cold tolerance [3]. The CBF pathway is present and cold-responsive in numerous plant species, including major tropical crop species such as soybean [4], rice [5], tomato [6], etc., and yet, when exposed to freezing temperatures, there is a marked difference in survivability between the cold adapted temperate and the tropical plant species [7]. Concurrently, studies have reported that overexpression of the CBF gene(s) increases the cold tolerance in *A. thaliana* [8], *Brassica napus* [3], poplar [9], and potato [10], but not in tomato [11]. More recently, the existence of a CBF-independent cold response regulation pathway has also been reported [12–17] indicating that the CBF regulon is co-regulated by other early cold-regulated transcription factors, thus increasing the complexity of the low temperature GRN.

Plants resist damage from low temperatures by accumulating small metabolites, such as soluble sugars and polyamines, called cryoprotectants. Many cryoprotectants act as osmoprotectants or stabilize membrane fluidity [18]. Glucose, raffinose, trehalose, and sucrose accumulate in cold-tolerant plants under stress and act as osmoprotectants [18–20]. Trehalose in particular, is an effective osmoprotectant [21]. Over-expression of trehalose biosynthesis genes have shown to improve stress tolerance in many plant species [22, 23], but the regulation of trehalose metabolism during stress conditions remains understudied. Similarly, raffinose accumulates in many plant species upon exposure to low temperature [24]. Polyamines (PAs) are another class of osmoprotectants that can bind to the phospholipid sites in the cell membrane to prevent cell lysis and improve cold tolerance [25]. The polyamine synthesis pathway gene, *S-adenosylmethionine decarboxylase (SAMDC)* is involved in biosynthesis of putrescine, spermidine, and spermine [26]. Overexpression of *SAMDC* resulted in increased tolerance to freezing in the centipede grass [27].

The plant circadian clock integrates inputs from the diurnal and seasonal changes in light, temperature, and other environmental factors [28]. Therefore, the plant transcriptional response to low temperature is highly variable depending on the time-of-day, a process called “clock gating” [29, 30]. Therefore, studies of transcriptional response to cold are often confounded by the crosstalk between the cold signal and the circadian clock [31]. The confounding effect of circadian regulation can be avoided by conducting studies in plants grown for long periods in continuous light (>10 days; circadian oscillation severely dampens due to lack of light entrainment) [32–34] or by attenuating the amplitude of clock oscillation via low-light entrainment. However, results from such studies may prove difficult to translate to field crop species such as tomato.

Despite the existence of a cold-responsive CBF pathway, tomato plants suffer cold injury at temperatures below 10°C [35] and were long considered unable to cold-acclimate [11]. However, recent studies [6] indicate that tomato may be capable of cold-acclimation albeit at a higher acclimation temperature (10°C) than *Arabidopsis* (4°C). Little is known about the early tomato response to cold. Finally, most of the cold-response studies have involved transcriptome profiling of plant aerial tissues including shoot, leaves, cotyledons, seed and fruit [36] with the inclusion of root tissues being rare in *Arabidopsis* [37], *Zea mays* [38], and in sugarcane [39], or none in tomato. The primary regulators of the root cold-response remain unknown, although the metabolomic response to cold stress in root indicates induction of oxidative stress by changing the levels of reactive oxygen species (ROS) and other metabolites [40].

**Figure 1:**
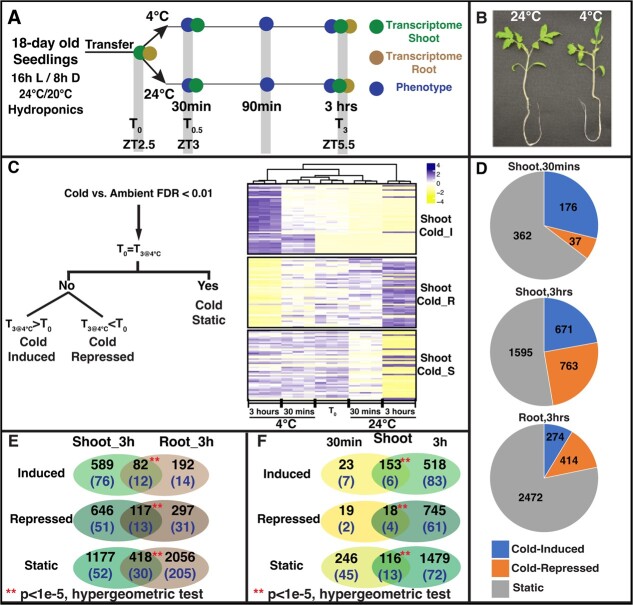
Transcriptional response to chilling stress is largely driven by disruption of rhythmic gene expression. **A.** 18-day old tomato seedlings were exposed at ZT2.5 to chilling stress and phenotyped at 30 minutes, 90 minutes, and 3 hours. Shoot transcriptome was sampled at 30 minutes and 3 hours post treatment, while root was sampled only at 3 hours. **B.** Three hours of exposure to 4°C induced severe wilting in tomato seedlings. These seedlings were nearly identical in size prior to treatment. **C.** Differentially expressed (DE) genes (quasi-likelihood F-test, *P* < 0.01) between 4°C and 24°C were further classified into cold-induced (gene expression increased over pre-treatment level (T_0_)), cold-repressed (gene expression reduced from pre-treatment level) and cold-static (gene expression essentially same as pre-treatment). The heatmap shows expression patterns of the top 50 genes from each set in the shoot. **D.** DE gene sets increase over time in the shoot and the root has a higher overall number of DEGs. However, most DE genes are cold-static in each organ. **E.** Venn diagram showing significant but not complete overlaps between shoot and root responses at 3 hours, indicating a shared cold-stress response, but also organ-specific responses. Numbers in each zone indicate the total number of genes in that bin, and numbers in brackets indicate TFs included in each set**. F.** Venn diagram showing overlap of cold-stress response at 30 minutes and 3 hours after exposure to 4°C. Some genes show an initial transient response, but most genes show sustained response to cold. The transcriptional response to cold stress increases as the exposure continues.

This study aims to elucidate the early and organ-specific transcriptomic response to cold stress and investigate the interaction between cold and circadian regulation in a crop model with tropical origin—tomato. This study contrasted the shoot and root cold-treated transcriptome (4°C) to the transcriptome of a same time-of-day ambient temperature control (24°C) after 3 hours of treatment as well as a before-treatment control (T_0_). Contrasting the root response to the shoots from the same plants allows identification of shared and unique cold responses between the two major organs. We further expand the cold-response gene set by including a very early cold-treatment shoot sample (30 minutes after start of cold treatment; Fig. 1A) to capture the early and transient cold-responsive genes. Overall, this experimental design allows us to query three crucial aspects of cold stress response in a chilling sensitive species: (1) What are the cellular/physiological processes affected early by cold stress and what are the TFs that regulate them? (2) Is the cold stress response similar at whole plant level or are there organ-specific responses? (3) Given the previously described interaction between cold stress and circadian regulation, does the dual-control design allow distinction of genes irrefutably responsive to cold versus those whose perceived differential expression is caused by an interaction of their response and their diurnal/circadian cycling?

## Results

### A 3-hour chilling treatment triggers wide-spread transcriptional reprogramming and morphological stress responses in tomato seedlings

To determine the duration of cold stress required to cause visual stress responses in tomato, 18-days old tomato seedlings (var. M82) grown under 16 hr/8 hr day-night cycle (100 μmol m^−2^ s^−1^, 24°C/20°C) were transferred to a container with pre-chilled growth media (4°C), or an ambient control (24°C), at ZT2.5 (Fig. 1A; where ZT = time of onset of light). The chilling treatment of 4°C was chosen based on prior studies of cold stress response in tomato [6]. Plants were visually examined prior to the treatment to ensure all seedlings are of the same size and at same developmental stage, as well as at 30, 90, and 180 minutes after start of treatment (Fig. 1A). The seedlings showed minor leaf curling after 90 minutes and severe wilting after 180 minutes of cold treatment (Fig. 1B and Fig. S1). These observations confirm that the cold treatment for 3 hours at 4°C was sufficient to generate a noticeable stress phenotype in the chilling sensitive tomato plants. Under these growth conditions, the timing of the chilling treatment coincides with the morning (ZT2.5) to noon phase (ZT5.5) of the diurnal cycle.

To understand the molecular basis of the observed physiological changes, both in the aerial tissues and in the underground tissues, shoots and roots were collected at (T_0_), i.e. just prior to cold exposure, as well as 3 hours after the start of cold treatment (4°C vs 24°C), for RNA-Seq analysis using the Illumina platform (Fig. 1A). For shoots only, to detect the early signaling components, tissue samples were collected 30 minutes after cold treatment (4°C vs 24°C). The generated RNA-seq data were first mapped to the reference tomato genome (ITAG v3.2) (Table S2) plus the organellar (chloroplast and mitochondrial) genomes to determine gene expression levels (see Methods). Next, differentially expressed genes (DEGs) in response to cold stress were determined using a quasi-likelihood F-test (edgeR [41]) between the ambient (24°C) and cold-treated (4°C) gene expression levels (at FDR < 0.01), at each time point (i.e. 30 mins to 30 mins and 3 hrs to 3 hrs) in the shoots and roots separately. After three hours of cold treatment, 3,864 genes in the shoots and 9,394 genes in the roots were differentially expressed between plants at 4°C and at 24°C (Table S3). Even as early as 30 minutes after cold treatment, 575 genes show expression level changes in 4°C compared to 24°C (Table S3). The shoot DEGs at 3 hours significantly overlap with the previously described cold-responsive tomato genes [6] (Table S7 and Fig. S2), but this study also identifies root specific DEGs as well as early responsive DEGs (30 minutes). Overall, our results revealed a widespread transcriptional reprogramming in the shoots as well as in the roots when tomato seedlings are exposed to chilling temperature.

### Tomato transcriptome response to low temperature (4°C) includes severe dampening of circadian/diurnal amplitude

A unique experimental component of this study is the profiling of the transcriptome at T_0_ to determine the temporal characteristics of the transcriptional response to chilling stress. Surprisingly, we observed that the majority of DEGs (4°C vs 24°C) showed a pattern of unchanged expression relative to T_0_*only* in cold. Specifically, we compared the gene expression level of DEGs at later timepoints (either 30 min or 3 hr) against T_0_, under cold treatment and ambient temperature separately, and classified the cold-responsive DEGs into three categories (Fig. 1C; see methods for details): (i) *cold-induced DEGs* (referred to as “*induced*” set) whose expression level is increased compared to T_0_ under cold treatment (T_0.5_ > T_0_ or T_3_ > T_0_ under cold treatment for shoots, and T_3_ > T_0_ under cold treatment for roots); (ii) *cold-repressed DEGs* (referred to as “*repressed*” set) whose expression level is reduced compared to T_0_ under cold treatment (T_0.5_ < T_0_ or T_3_ < T_0_ under cold treatment for shoots, and T_3_ < T_0_ under cold treatment for roots); and (iii) *cold-static DEGs* (referred to as “*static*”) whose expression level is unchanged compared to T_0_ (T_0.5_ ≈ T_0_ or T_3_ ≈ T_0_ under cold treatment for shoots, and T_3_ ≈ T_0_ under cold treatment for roots). By contrast, the mRNA levels of cold-static genes differ from T_0_ in ambient conditions (24°C), presumably due to circadian and/or diurnal regulation. In addition, DEGs whose expression fold-changes (T_0.5_/T_0_ or T_3_/T_0_) was mild in both 4°C and 24°C samples were labeled “indeterminate” and left out of subsequent analyses. The cold-responsive DEGs identified at 30 min in shoots include 176 *induced*, 37 *repressed* and 362 *static* genes. At 3 hr, the corresponding induced, repressed and static set sizes for shoots are 671, 763, and 1595, with the static set being the largest group. There are highly significant overlaps between the gene sets regulated in the same direction (i.e. induced vs. induced, repressed vs. repressed and static vs. static) at 30 min and 3 hr (Fig. 1F). For roots, we observed an even bigger proportion of static genes, with 274 induced, 414 repressed, and 2,474 static genes (Fig. 1D; Table S3). There is a significant overlap but also distinct DEGs between shoots and roots (Fig. 1E). There were minimal overlaps between genes induced at 30 minutes and repressed at 3 hours or vice versa (Fig. S3). Minimal overlaps (four genes) were found between the shoot induced and root repressed (and vice versa) gene sets (Table S3), implying that genes are either monotonically induced or repressed by cold stress across the 3-hour treatment and across organs.

To gain insights into the biological processes enriched in the shoot and root DEGs, GO functional analysis was performed for the induced, repressed, and static genes identified. Cold stress, in shoots, significantly affected numerous biological processes in the cold-induced DEGs and the cold-static gene set but a smaller number of processes in the cold-repressed DEGs (Table S14 &S16). Cold stress induced a large set of genes belonging to photosynthetic process, response to water deprivation, response to cold etc. whereas the mRNA and mitochondrial RNA modification processes were significantly repressed in shoot tissues. In the static gene set processes related to primary metabolism were affected by cold stress (Fig. S4; Table S14,S16). We further classified the cold static geneset into two subsets: (i) induced relative to 24°C (static_induced) and (ii) repressed relative to 24°C (static_repressed) and performed GO enrichment analysis on each set. This subset approach revealed that almost all the GO enrichment seen in the cold static set, at both 30 minutes and 3 hours, is due to the static_induced genes while the static_repressed genes were not enriched for any GO process (Table S14 andS16). In tomato roots, the genes induced by cold stress were not significantly enriched in any of the GO categories, however cold stress affected numerous primary metabolism processes in the cold-repressed gene set (Table S17 andS18; Fig. S5), while the cold static gene set was enriched for response to oxidative stress, response to water deprivation, response to hormone, etc. (Table S18).

**Figure 2:**
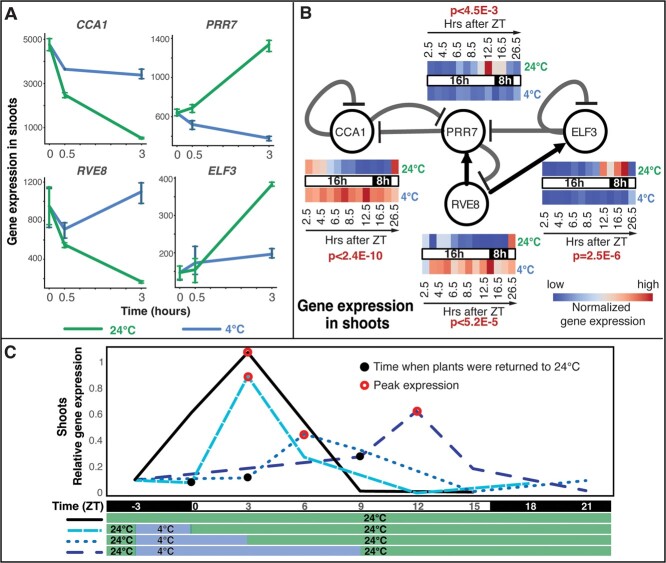
Amplitude of oscillation is severely dampened for multiple core clock genes and resumes hours after return to ambient temperature. **A.** Chilling stress disrupts the cycling of various core clock components. Shown are expression level changes of four core clock genes in ambient temperature (green line) and under chilling stress (blue line). These genes are classified as cold-static. (Note: All gene names shown are derived from Arabidopsis orthologs.) **B.** A selected set of four clock genes show characteristic oscillation of gene expression in a 24-hour cycle, under ambient conditions (24°C, 16 h L/8 h D) (heatmap above the light conditions bar for each gene). A concurrent chilling treatment (also 24 hours) reveals that the amplitude of oscillation remains dampened for these core clock genes during the entire 24 hours of chilling treatment (heatmap below the light conditions bar). Specifically, there is no observable change in expression level for CCA1, PRR7, ELF3 during the 24 hours in 4°C. P-values (in red) are from testing for significant difference in a fit for cubic spline-based regression fit between the ambient and cold-treated expression trajectories. **C.** The core clock morning gene, CCA1 reaches peak expression (red circles) 3 hours after ZT in ambient conditions. Under cold treatment, CCA1 expression remains flat for the duration of cold and reaches peak approximately 3 hours after plants return to 24°C (black dots). Note: Data shown in panels B &C was generated by multiplexed amplicon sequencing from cDNA libraries (see Methods).

The differential expression of the dominant “static” set of genes is possibly caused by mis-regulation of core clock components during cold stress. A previous study has shown that cold stress severely dampens the amplitude of clock gene oscillation in Arabidopsis [29]. Therefore, we investigated the expression patterns of core clock genes under cold stress in tomato. In the shoots, our results show that under ambient temperature, the mRNA level of the tomato homolog of *CCA1*, the central morning-phased clock gene, is reduced by 10-fold from T_0_ to T_3_ (from ZT2.5 to ZT5.5). By contrast, in the cold-treated samples, the repression of *CCA1* is greatly attenuated and its mRNA level is reduced by only 30% from T_0_ to T_3_ (Fig. 2A; Table S5). Two other morning phased genes *RVE6* and *RVE8* are similarly reduced by 2-fold and 5-fold in ambient conditions, but not under chilling conditions (Fig. 2A andS6; Table S5). Conversely, induction of evening clock components such as *PRR7* (2-fold), *GI* (8-fold) and *ELF3* (2.5-fold) observed in ambient conditions failed to occur in the cold-treated samples (Fig. S6; Table S5). Finally, aberrant induction of another clock component *PRR9* (2.5-fold) was seen only in the cold-treated samples. Similarly, in the roots, the repression of *CCA1* (4-fold) and *RVE8* (2-fold), and the induction of *PRR7* (1.6-fold), *GI* (1.5-fold), *LUX* (6-fold) and *TOC1* (2-fold) that is seen in ambient conditions is absent in the cold-treated plants while aberrant induction of *PRR9* (2-fold) is observed (Fig. S7; Table S5). The changes in core clock gene expression levels seen in the RNA-Seq data were validated using quantitative reverse transcription (qRT-PCR) (Table S6; Fig. S6).

To extend our observation of the effects of chilling on clock gene oscillations from the original 3 hr to a longer 24 hr period, we performed an additional experiment at ZT2.5 that placed tomato seedlings in either 4°C or 24°C for a 24-hour period and sampled them at T_0_, 1, 2, 3, 6, 12 and 24 hours. Expression levels of four representative clock genes: *CCA1*, *PRR7*, *RVE6* and *ELF3* were measured from each time point under ambient and cold conditions. In cold, the expression levels of these four genes remained essentially unchanged (Fig. 2B). In plants that remain at 24°C these genes show a diurnal maxima and minima in gene expression as expected (Fig. 2B); at 24°C, *CCA1* expression level is highest at ZT2.5 and again 24 hours later, while the *PRR7*, *ELF3* and *RVE8* maxima are at ZT14.5, ZT18.5 and ZT26.5 respectively. In all four of the clock genes measured, the amplitude of oscillation in gene expression is dampened at 4°C (Fig. 2B; Table S22).

**Figure 3:**
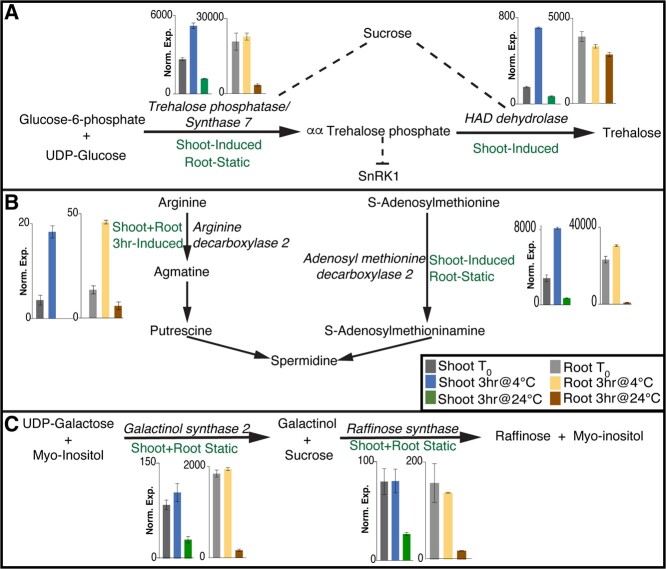
Chilling stress induces expression of cryoprotectant biosynthesis genes in both shoots and roots. Net effect of these changes would be accumulation of these cryoprotectants under chilling stress, as previously shown (Barrero-Gil et al., 2016). All genes shown here were induced between 4°C and 24°C in both the shoot and root (FDR < 0.01). **A.** Enzymes for both steps in trehalose synthesis are expressed higher under 4°C in shoots and roots. **B.** The two committing steps in Polyamine biosynthesis are expressed higher in 4°C in both organs. **C.** Raffinose biosynthesis genes are expressed at higher levels at 4°C in both shoots and roots.

To determine the time taken for resumption of a normal amplitude of clock oscillation after the plant returns to ambient conditions, we investigated the level of expression of CCA1 for multiple hours after the end of cold treatment. CCA1 expression levels were assayed 3, 6, 9, and 12 hours after varying lengths of cold treatment. In the control plants, with no cold treatment, CCA1 reaches peak expression 3 hours after ZT (black, solid line in Fig. 2C). Plants in the treatment group were exposed to either 3, 6, or 9 hours of cold (4°C) before being returned to ambient temperature (24°C). Irrespective of the time of day, the expression level of CCA1 reached its peak at 3 hours after the end of cold treatment (blue, dotted lines in Fig. 2C). Thus, the temperature experienced by the plant had a stronger effect on CCA1 expression than the incidence of light (ZT), which was the same for all plants (Fig. 2C).

To determine whether the cold-static gene set is normally under the control of diurnal or circadian regulation, these gene sets were compared with genes undergoing diurnal or circadian cycling [42]. The tomato cold-static genes were mapped to their closest Arabidopsis ortholog using the OrthoFinder algorithm [43], and then subsequently overlapped with the known Arabidopsis diurnal (LDHC) and circadian (LL_LDHC) gene sets from the Diurnal database [42]. Indeed, the cold-static gene set has highly significant overlaps with the gene sets shown to be diurnally regulated under long day (hypergeometric test, *P* < 6e-15) (Fig. S8) or continuous light (hypergeometric test, *P* < 7e-29) (Fig. S8) [42]. Overall, our data showed that during cold stress, the amplitude loss of core clock genes likely disrupts the rhythmic regulation of downstream genes, observed as the large cold-static groups in the shoots (53%) and the roots (78%). Therefore, cold stress seems to include a direct response (cold-induced and repressed) that may be more directly related to the mitigation of stress, as well as an indirect response (cold-static set) that may be downstream or incidental. Thus, all further analyses treated these gene sets separately.

**Figure 4:**
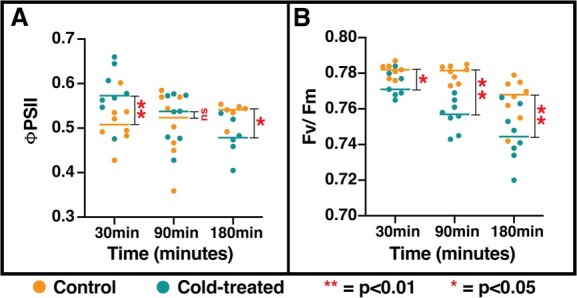
Photosynthetic efficiency of tomato plants is sharply reduced within 3 hours of exposure to 4°C. **A.** The operating efficiency of photosynthesis (ΦPSII) is initially (30 minutes) enhanced in the cold-treated samples (*t*-test, *P* < 0.05). As the cold treatment continues, ΦPSII falls despite the sustained upregulation of associated genes (*t*-test, *P* < 0.05). **B.** The maximum efficiency of photosynthesis, a proxy for PSII health, falls continuously through the cold treatment (*t*-test, *P* < 0.05). Thus, the remarkable upregulation of most photosystem genes fails to compensate for the net loss of photosynthetic efficiency caused by chilling stress in the timescale we examined.

### Cold induces the synthesis of known osmoprotectants in both shoots and roots

Accumulation of compatible solutes with cryoprotective properties, such as proline, sucrose, trehalose and raffinose [44, 45] and secondary metabolites such as polyamines (PA’s) [25, 46] have been shown to have a significant role in enhancing the cold tolerance in plants. While their roles in protecting the aerial tissues against cold stress is well known, it remained a question whether the underground tissues (roots) also employ similar strategy in cold response. Our genome-wide study showed that chilling stress induces multiple genes involved in the biosynthesis of trehalose and polyamines within 3 hours of exposure to cold in both shoots and roots (Fig. 3A and B). Overall, the mRNA levels of genes catalyzing trehalose, raffinose and polyamine biosynthesis are, on average, 10-fold higher in the cold treated plants than the ambient plants, yet the specific regulation pattern could be distinct between shoots and roots.

Trehalose is a disaccharide synthesized in a two-step reaction from glucose-6-phosphate and UDP-glucose. The first step generates αα-trehalose-phosphate and the second step cleaves the phosphate group to release the trehalose disaccharide. In the shoots, genes encoding the enzymes for the first (Trehalose phosphatase/Synthase 7 *Solyc02g072150*) and second step (HAD dehydrolase, *Solyc04g072920*) were induced compared to T_0_ within 3 hours of chilling stress (i.e. “cold induced”; see Fig. 3A). In the roots, the expression of both these genes was retained at the T_0_ level in 4°C (cold-static) but declined in ambient conditions (Fig. 3A) with a net effect of elevated mRNA levels in 4°C relative to 24°C. Therefore, although the trehalose pathways are up-regulated in cold treated samples compared to ambient controls in both shoots and roots, their specific regulatory mechanism, especially in relation to the clock control, could be different in different organ. Similarly, polyamine synthesis from arginine and S-adenosylmethionine involves two committed enzymatic steps (Fig. 3B) and genes encoding enzymes for both steps (Arginine decarboxylase 2; *Solyc01g110440* & Adenosyl methionine decarboxylase 2; *Solyc02g089610*) were induced within three hours of chilling stress in the shoots. In the roots the gene encoding the enzyme for the first step (Fig. 3B) is induced within 3 hours, while the diurnal repression of the second step is suspended by chilling stress. Finally, raffinose is another potent cryoprotectant and its biosynthesis also involves a two-step process. During chilling stress applied in this study, the mRNA levels of genes encoding both these steps (Galactinol synthase 2; *Solyc02g084980* and Raffinose synthase; *Solyc02g086530*) are retained at the same level as T_0_ in 4°C but decline in 24°C (i.e. cold-static) in both the shoots and roots (Fig. 2C). Previous studies in Arabidopsis [47], Tomato [6], Rice [48] etc. have demonstrated increased accumulation, albeit on longer time scales, of these cryoprotectants in response to cold stress.

### Functional and pathway enrichment analyses of cold-responsive genes highlight early effect of cold stress on photosynthesis

To identify functional enrichment among the cold-responsive DEGs, Kyoto Encyclopedia of Genes and Genomes (KEGG) pathway enrichment analysis was performed. The analysis revealed that many genes involved in photosynthesis, hormone signaling etc. were induced by cold stress. In roots, no pathway enrichment was observed in the cold-induced gene set. The genes in the static set were found to be enriched for numerous metabolic pathways such as plant-pathogen interactions, phenylpropanoid biosynthesis, signal transduction, etc.

**Figure 5:**
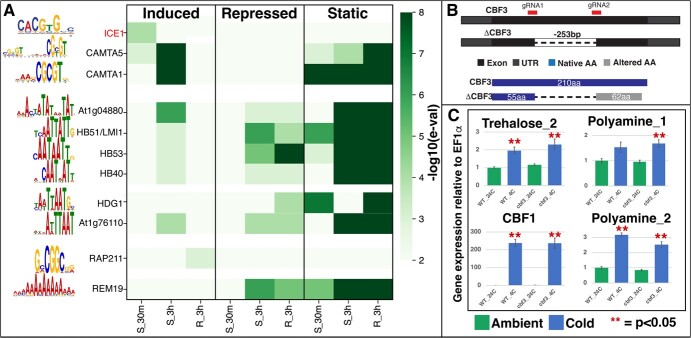
Gene sets classified by time and organ show evidence of distinct regulatory motifs. A. Promoter analyses identifies conserved motifs that are overrepresented in induced, repressed, and static gene sets. Distinct conserved motifs are seen in each organ and timepoint. The earliest cold-induced gene set (shoot 30 minutes) is enriched for the binding sites of CAMTA5, and a bHLH family binding motif. B. A knockout line of CBF3 was generated via CRISPR/Cas mutagenesis. C. CBF1 and cryoprotectant biosynthesis genes are induced by cold treatment even in the lack of CBF3. A 2-way ANOVA test with treatment and genotype as factors revealed significant differences due to treatment, but not genotype (*P* < 0.01) for all four genes tested.

The most significantly affected process/pathway in the shoots of tomato plants under cold stress is the photosynthesis pathway. Specifically, as the tomato shoot experiences cold stress, it increases the expression of many components of photosystems I and II as well as cytochrome *b*_6_*f* complex within 30 minutes (Fig. S9). Further induction of expression of these genes was observed up to 3 hours after start of cold stress (Fig. S9). Strikingly, all these induced genes are plastid encoded expect for Fd (ferrodoxin) in the electron transport system (Fig. S9). The observation of induction of genes in the photosynthetic pathway contrasts the previously described cold-induced reduction in photosynthetic efficiency in *Arabidopsis* [49, 50] and Tomato [51]. Therefore, to determine the net effect of cold stress on plant photosynthetic rate, photosynthetic rate parameters (specifically ϕPSII, F_v_/F_m_) were measured for plants in control conditions (24°C) as well as cold-treated (4°C) for 30, 90 or 180 minutes. The fluorescence parameter ϕPSII (PSII operating efficiency) and F_v_/F_m_ (PSII maximum efficiency) were previously shown to be altered in plant cold response [52]. The maximum efficiency of PSII (measured as F_v_/F_m_) decreased significantly, indicating damage to the PSII in the cold-treated plants at all timepoints after plant exposure to cold stress (Fig. 4; Table S20). Comparing the ϕPSII between the before and after cold-treatment plants showed a slight increase in ϕPSII at 30 minutes (0.51 vs 0.58, *P* < 0.01), followed by a reversion to parity at 90 minutes (0.51 vs 0.52, *n.s.*), and then a significant reduction (0.53 vs 0.46, *P* < 0.05) after 180 minutes at 4°C. In agreement with the FPSII measurements, the linear electron flow through PSII also showed a significant increase at 30 minutes, a reversion to parity at 90 minutes and a significant reduction at 180 minutes (Table S20; Fig. S10). Thus, the net effect of chilling on photosynthetic efficiency is negative despite the rapid and robust increase in expression levels of genes encoding PSI and PSII components (Fig. S9).

### Transcriptional regulation of cold-stress response in shoots and roots

The CBF family of TFs have been well characterized as regulators of cold stress response in *Arabidopsis* [53] and to a lesser extent in tomato. In this study, *SlCBF3* (Solyc03g026280), was induced in both shoots and roots, *SlCBF2* (Solyc03g124110) is induced in the shoots and is static in the roots, and *SlCBF1* (Solyc03g026270) (see Fig. S11 for nomenclature) was static in the shoots and repressed in the roots. Orthologs of some of the TFs previously identified as first wave transcriptional regulators of cold response [54] were up regulated in tomato shoots, e.g. *ZAT10*, *WRKY33*, *ERF5*, *WRKY22*, *ZAT12*, and *WRKY40*. However, in the roots these TFs fall into the cold static group, indicating that these TFs might have tissue-specific regulatory roles in response to cold stress (Table S4). Indeed, many of the cold-responsive TFs (53%–83% across categories) are organ-specific, in agreement with overall DEG sets (Fig. S12). The overlap of cold-responsive TFs between the organs suggests that a core cold-responsive GRN is active in both organs. However, the secondary TFs and hence the cold-signal transduction may be substantially different in roots and shoots of the same plant. A selected set of commonly induced or repressed TFs from RNA-Seq analysis were validated by qRT-PCR studies from the shoot and root tissues (Fig. S13; Table S9).

To investigate the early transcriptional cascade in response to cold, we further analyzed the transcriptional response at 30-mins of cold treatment in the shoots. Among the 575 genes that are differentially expressed between 30 min of cold treated and ambient control plants, 77 genes encode TFs. Of the first wave cold-responsive TFs in Arabidopsis [12], only *SlCBF3*, *SlCBF2*, and *SlZAT10* were identified in our 30-mins cold-treated samples, indicating differences in the early transcriptional cascade of cold stress response between these two species (Table S10). Indeed, we identified novel TFs from the tomato dataset to be early cold responsive regulators including two LBD family TFs namely, LBD37, LBD41, and an HSF family TF, HSFC4A, providing candidates for further studies to elucidate the precise role of these TFs in cold stress response. These observations from RNA-Seq were confirmed by qRT-PCR studies from the same tissue (Fig. S13; Table S11).

To establish whether the early responsive TFs (30 min) regulate the late responsive DEGs (i.e., 3-hour DEGs), the cold induced, repressed and static gene sets in the shoots were subjected to *cis*-motif analyses to identify enriched TF binding sites in the promoters of each set of genes (Table S12; Fig. 5A). The early (30 minutes) cold induced genes showed an enrichment of binding sites for CAMTA5, multiple bHLH TFs, as well as multiple BZR1 homologs, implying that the early cold induced genes are regulated by a combination of CAMTA, bHLH family and Brassinosteroid responsive TFs (Table S12). The promoters of the cold-induced genes at 3 hours are enriched for CAMTA 1/5 binding sites as well as multiple Homeobox family TFs (Fig. 5A). The cold-repressed set of genes show enrichment of the binding site for IDD4, NGA4 and TCP3 at the 30-minute time point and enrichment of the binding site for the Homeobox family and REM19 at the 3-hour time point. Finally, the promoters of the cold-static gene set are enriched in binding sites for the CAMTA TFs at 30 minutes and Homeobox family of TFs at 3 hours after col treatment (Table S12).

Prominently missing from the motif enrichment results is the known binding site for the CBFs, which was previously implicated in the induction of cryoprotectants (e.g. trehalose, polyamines) [55]. We hypothesized that the CBF pathway is not necessary for the observed induction of biosynthesis genes for trehalose and polyamines (Fig. 3). We tested this hypothesis by first generating a knock-out mutant for the strongest induced CBF, CBF3 (shoots FC = 400 and roots FC = 155) and then exposing a wild-type plant and the *Δcbf3* (Fig. 5B) to 4°C for 1.5 hours. The *Δcbf3* allele used here is missing 253 bp, including the portion coding for the core AP2/ERF domain and is predicted to generate a highly truncated protein (Fig. 5B). The expression level of CBF1 and cryoprotectant biosynthesis genes was then assayed in both plants. CBF1 expression was induced in both the wild-type and Δcbf3 lines (Fig. 5C) implying the cold signal to CBF1 is maintained in the mutant. In both the wild-type and *Δcbf3* lines trehalose and polyamine biosynthesis genes were induced at approximately the same fold-change (Fig. 5C), implying that a functional CBF3 is not required for their induction in cold-stress response.

The promoters of the root cold-induced gene set are enriched in binding sites for EP2, bZIP and MYB families of TFs (Table S12; Fig. 5). Conversely, the promoters of root cold-repressed and cold-static gene sets are enriched with binding sites of a wide range of TFs such as the REM, ARID, Homeobox, CPP families. As observed in the cold-responsive TFs, there is significant similarity (e.g. REM19 and AT1G76110 motifs in repressed sets, Table S12) but also large differences in the cis-binding motif sets between the shoots and the roots.

## Discussion

The molecular components of cold response, acclimation and tolerance have been extensively, albeit incompletely, elucidated in the temperate model plant *A. thaliana* [53]. Tomato serves as a good model to investigate the cold stress response in tropical, agronomically important plants that are sensitive to cold stress. This study investigates two crucial, but understudied aspects in cold response in a sensitive species: 1. The early transcriptional cascade in response to cold and 2. The organ specificity of the cold response by separately profiling the transcriptome response in the shoots and the roots.

**Figure 6:**
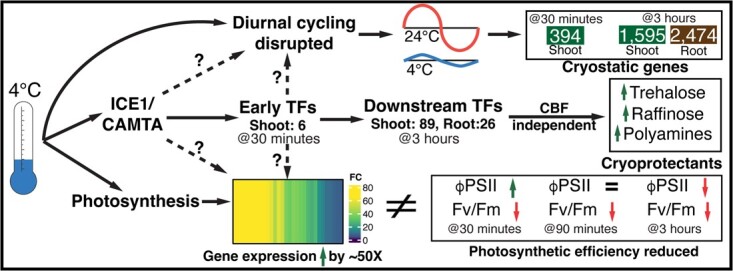
Chilling stress dampened oscillation of core clock components and downstream genes, reduced photosynthetic rate and elicited a transcriptional cascade in tomato seedlings. Rhythmic expression of core clock genes is disrupted either via ICE1/CAMTA factors or directly by the unknown cold sensor, leading to misregulation of a set of cryo-static genes that constitute a majority of cold transcriptional response. The TF cascade in response to cold starts small with 6 TFs induced within 30 minutes and expands to 89 TFs within 3 hours in the shoot. The first set of cold-responsive genes (i.e. within 30 minutes) are regulated by CAMTA and ICE1 TFs which themselves are not transcriptionally regulated. The root cold responsive TFs, even at 3 hours are far fewer at 26. In both organs, this transcriptional cascade leads to the increased biosynthesis of cryoprotectants in a CBF independent manner. Chilling stress has a negative effect on photosynthetic efficiency, despite the plant’s effort to compensate by increased expression of photosystem I and II genes. FPSII increases initially (30 minutes) then levels off and finally drops under chilling stress. Fv/Fm drops continuously during chilling stress leading to lower photosynthetic efficiency.

Previous studies have shown that the initial stages of signal transduction in cold stress signaling are primarily post translational [56–59]. Based on our motif analysis, the initial transcriptional changes in Tomato are likely driven by a pair of CAMTA TFs and a bHLH family TF (Fig. 5). The most likely candidate for the bHLH family TF is ICE1 which plays a key role in cold stress response [53]. Similar to *Arabidopsis*[56–58], neither the CAMTAs nor ICE1 respond transcriptionally to chilling stress. Curiously, the BZR1 binding site (Table S12) is enriched in the cold-induced genes within 30 minutes. BZR1 has been shown to regulate cold tolerance via CBF dependent and independent pathways in *Arabidopsis* [60]. Although the BZR1 motif is enriched in the 30-minute cold-induced set, which includes many photosynthesis genes, our data cannot establish the timing of this regulation. Dynamic TF-DNA binding studies are needed to establish the early regulatory role of BZR1 in cold-induced regulation of photosynthesis genes. A recent study showed that BZR1 accumulation protects from cold-induced photoinhibition by inducing an increase in levels of apoplastic hydrogen peroxide accumulation, which in turn leads to an induction of PGR5 dependent cyclic electron flow in the plastids of Tomato seedlings [51]. BZR1, thus provides a possible regulatory link between the early response to chilling stress and the downstream management of photoinhibition observed in cold stress [51]. The canonical ICE1 ➔ CBF ➔ COR pathway described in *Arabidopsis* is at least partially functional in Tomato shoots. SlCBF3 (Solyc03g026280) & SlCBF2 (Solyc03g124110) are induced within 30 minutes and remain induced at 3 hours, while SlCBF1 (Solyc03g026270) is in the shoot cold-static group. In the roots, at the 3-hour time point, SlCBF3 is induced, SlCBF2 is in the cold-static group and SlCBF1 is repressed. However, the CBF binding site is NOT over-represented in the 30-minute shoot or the 3-hour shoot or root gene sets. Our knock-out analysis of CBF3 (Fig. 5B and C) shows that the induction of cryoprotectant biosynthesis occurs even in the absence of a functional CBF3, even though CBF3 is heavily induced (Table S4). Thus, even though the CBF genes are induced, their role in the regulation of the cold response may be more limited in Tomato, at least under the rapid chilling stress applied in this study. Importantly, despite these similarities to Arabidopsis in cold response, Tomato has a much lower tolerance of chilling stress. The differences are likely due to the downstream transcriptional cascade and hence the processes affected. Our study samples the transcriptome response at 3 hours and identified 89 TFs in shoots and 26 TFs in roots that are induced within 3 hours with only 12 shared between them. This study establishes the modest set of TFs (6 induced) involved in the early cold-responsive transcriptional cascade that leads to the activation of protection mechanisms such as the synthesis of cryoprotectants (Fig. 3). Despite the accumulation of such cryoprotectant molecules (Fig. 3) [6], tomato plants are much more sensitive to chilling damage than *Arabidopsis*, suggesting that these metabolic changes are either quantitatively different between the two species or insufficient to alleviate the damage caused by low temperatures. Further elucidation of the roles of each of the cold-responsive TFs is needed to understand the structure of the full transcriptional network and to establish strategies to improve cold tolerance in tomato.

Our study captures the molecular response of roots to sudden chilling. We identified a set of 70 TFs that are induced or repressed (*P* < 0.01, FC > 2) in the roots due to cold stress (Fig. 1D). Promoter cis-motif enrichment analysis however identified few TFs that may regulate the root cold-induced genes, likely due to a lack of knowledge of the binding sites of these TFs. The relatively late 3-hour time point sampled in the roots prevents the identification of early responsive TFs, but the lack of CAMTA binding site over-representation in the cold-induced genes again suggests that the master regulators of root cold stress response are distinct from the well-established CAMTA/ICE1 ➔ CBFs ➔ COR pathway in *Arabidopsis* shoots.

Our study shows that chilling stress leads to the mRNA level of a set of genes being stabilized at the pre-exposure levels. These genes are identified as differentially expressed solely because their mRNA levels either increase or decrease in the same time frame under ambient (24°C) conditions. This set of genes is labelled cold-static genes in our study. In fact, the cold-static gene sets constitute the majority of the differentially expressed genes in the shoots (*n* = 1595) and roots (*n* = 2474) (Fig. 6). However, many genes continue to show diel oscillation under chilling conditions (Fig. S18) suggesting that some downstream effects of the clock continue to operate in the 3 hours after start of chilling stress. Previous studies have shown the gating effect of the circadian clock on the cold responsive transcriptome [29] as well as the input of the cold stress on the clock [61] e.g. CBF1 regulation of LUX1 [62]. A recent study demonstrated that multiple abiotic stresses could induce phase advances in specific clock components in Soybean [63]. In our study, chilling stress caused robust changes, relative to the ambient control, in the expression levels of CCA1, RVE6/8, PRR7/9, GI and ELF3 in the shoots AND in the expression levels of CCA1, RVE8, PRR7/9, GI, LUX and TOC1 in the roots. Further, we show that the amplitude of oscillation of four clock genes (CCA1, PRR7, ELF3 and RVE8) is severely dampened in chilling conditions (4°C) for at least up to 24 hours. Finally, using the expression level of CCA1 as a proxy, we show that the clock genes resume oscillation within 3 hours of return to ambient conditions and that this time frame of resumption is not dependent on the length of the cold treatment (Fig. 2C). While these changes in the specific clock component expression do not automatically prove a phase change or complete disruption of the circadian clock, the concurrent stasis of mRNA levels for thousands of cold-static genes (Fig. 1C and D) in both organs suggests a disruption in their diurnal regulation. Therefore, it is very likely that sets of cold-responsive genes identified via conventional diurnally coupled sampling conflates the truly cold-responsive genes, i.e. genes affected by the cold-sensing signaling and transcriptional cascades, with the set of genes whose diurnal rhythm is disrupted (Fig. S14). Further, a similar confounding effect is likely to have occurred in all gene expression studies examining any stress that modulates the circadian clock [63]. With the dual controls described here, it is possible to isolate genes that show a clear induction or repression in response to cold (Fig. 2; Table S3), or indeed other abiotic stresses. Such genes may be stronger candidates to influence stress tolerance since their expression level is directly regulated by the stress-responsive transcriptional cascade.

Our transcriptomic profiles from the 30-minute and 3-hour samples identified that most of the component genes of photosystem II were induced at both these time points (Fig. S9). The photosynthetic parameter measurements show that the maximum efficiency of PSII (measured as F_v_/F_m_) drops during the cold stress period (Figs 4 and6). Previous studies have shown that the intrinsic D1 protein which forms the core of the PSII electron transport system is sensitive to low temperatures [64]. Tomato seedlings seem to respond to the cold-induced damage to photosynthetic efficiency by ramping up the expression of photosynthetic genes (Fig. S9). We observe a transient increase in the operating efficiency of photosystem II (ΦPSII) within 30 minutes of cold stress. Despite the rapid induction of photosynthetic genes, cold stress leads to a steady decline in the maximum efficiency of PSII measured as F_v_/F_m_ (Fig. 4; Fig. 6) over the three-hour time frame. The inability, of increased transcription of PSII components (Table S24), to rescue photosynthetic efficiency may be explained by two underlying reasons: *i.* D1 protein translation rate is reduced due to ribosomal pausing in low temperatures [65] and *ii.* the molecular chaperone (CDJ1) needed for proper folding of D1 protein is not induced by cold until 6 hours post treatment [66] and hence D1 folding is impaired in the initial time frame of 3 hours investigated here.

When the rate of PSII damage exceeds the rate of repair photosynthetic efficiency is reduced [67, 68]. Thus, these plants do not benefit from the energy spent to shore up photosynthesis during the initial phase of cold stress. Photosynthesis involves the unavoidable production of ROS, which should promptly be scavenged for safe and efficient light energy conversion. The cold-induced damage to photosystems could exacerbate the production of ROS, which may exceed the capacity of the scavenging enzymes. A recent study highlighted the role of cytosolic ROS in activating CBFs and enhancing freezing tolerance [59] which suggests a role for the plastids in rapidly amplifying the cold signal via rapid metabolite signaling. In fact, the cold tolerant varieties of Tomato were shown to increase their survival by expending energy to generate excess maltose that helps better mitigate the damage to PSII [69]. The combination of energy cost to maintain photosynthetic efficiency and prevent and repair ROS damage would be detrimental to the long-term growth of the plant and may thus, at least partially, explain the higher cold sensitivity of Tomato and other tropical plants.

## Materials and methods

### Plant material, growth conditions and cold treatment

Tomato seeds (*L. M82* cultivar, TGRC accession: LA3475) were sterilized with 20% bleach solution for 20 minutes, rinsed thoroughly with deionized water, germinated on 1/2MS-media in Petri dishes in a growth chamber set at 24°C/20°C (day/night) with a photoperiod of 16 h/8 h (day/night) and a PAR of 100 μmol m^−2^ s^−1^ for 7-days. Seedlings were then transferred to a hydroponics system containing Hoagland solution (Figs. S1 andS15). The solution was renewed every 3 or 4 days and the pots were aerated for 2 h/d, for 11 days. On the 18th day, tomato plants of nearly identical size were chosen and divided into two, equal groups and moved to fresh hydroponic containers 2.5 hours after the start of light cycle (i.e. Zeitgeber time (ZT) 2.5). One group remained at 24°C and the other group was transferred to 4°C in a cold-room. Both groups were exposed to light with a PAR of 100 μmol m^−2^ s^−1^. Each time-point includes three biological replicates per condition (4°C and 24°C). Each biological replicate is a pool of shoot or root samples from four seedlings. All samples were harvested in liquid nitrogen and stored in −80°C until RNA isolation for further analysis.

### RNA extraction from shoot and root tissues

Total RNA was extracted from 18 shoot samples (two treatments × two timepoints × three biological replicates + six time zero samples) and nine root samples (two treatments × three biological replicates + three time zero samples) with RNeasy Plant Mini Kit (Qiagen, USA) following the manufacturer’s protocol. The quality and quantity of RNA were checked using the Nanodrop 1000 spectrophotometer (ThermoScientific, USA). RNA integrity was assessed using the Bioanalyzer 2100 system (Agilent Technologies). All samples had RIN values >8. The RNA samples were processed, and sequencing libraries were generated using the Illumina TruSeq RNA Sample preparation kit for cDNA library preparation and sequenced on the Illumina HiSeq platform (150 cycles, paired end). Sequencing of the libraries was performed across 3 batches resulting in range of sequencing depth from ~23–120 million paired end reads (Table S2).

### Identification of cold-responsive genes

The RNA-Seq generated >30 million reads per sample. Reads were aligned to the ITAG 3.2 reference genome, plus the plastid and mitochondrial genomes, using BBMap (v37.93) [70] with default parameters and read counts calculated via the featureCounts package [71]. Read counts were then normalized using the TMM method in the edgeR package [41]. Agreement between biological replicates for each group was checked with an MDS plot (Fig. S16). Normalized counts were used to identify differentially expressed genes (DEG) between the cold-treated (4°C) and ambient treated (24°C) plants at the same time point, using a quasi-likelihood F-test [72] implemented in the edgeR package [41]. Significance threshold was set at an FDR corrected *P* < 0.01.

### Classification of cold-responsive genes into induced, repressed and static groups

For each DEG (FDR < 0.01) at each time point, the fold-change between the gene expression level at 4°C and its expression level at T_0_ was computed (FC(4vsT_0_)). For a cold-responsive gene to be considered cold-induced it’s FC(4vsT_0_) has to be > = 1.5 at 30 minutes or > = 2 at 3 hours. Conversely, for the gene to be considered cold-repressed it’s FC(4vsT_0_) has to be <= 2/3 at 30 minutes or < = 1/2 at 3 hours. If FC(4vsT_0_) does not pass these cutoffs, the fold-change between gene expression level at 24°C and T_0_ was then computed (FC(24vsT_0_)). If FC(24vsT_0_) passes these same cutoffs, then the gene is considered cold-static (see Fig 1C). If neither FC passes these cutoffs, then the gene is classified as indeterminate. Therefore, *cold-induced DEGs’* expression level is increased compared to T_0_ under cold treatment (T_0.5_ > T_0_ or T_3_ > T_0_ under cold treatment for shoots, and T_3_ > T_0_ under cold treatment for roots); (*ii) cold-repressed DEGs*’ expression level is reduced compared to T_0_ under cold treatment (T_0.5_ < T_0_ or T_3_ < T_0_ under cold treatment for shoots, and T_3_ < T_0_ under cold treatment for roots); and (*iii) cold-static DEGs’* expression level is unchanged compared to T_0_ (T_0.5_ ≈ T_0_ or T_3_ ≈ T_0_ under cold treatment for shoots, and T_3_ ≈ T_0_ under cold treatment for roots). Since each time point is considered independently, a gene may be classified into one group at 30 minutes and another group at 3 hours, implying a highly dynamic regulation of that gene. For identification of transcription factors, regulators and protein kinases encoding genes, the DEGs were searched against the iTAK database (v1.6) [73].

### Comparison of circadian and diurnal datasets

The cold-static gene set from tomato was mapped to its orthologs from Arabidopsis using the OrthoFinder algorithm [43] using the basal angiosperm *Amborella trichopoda* as outgroup. The Arabidopsis diurnal (LDHC) and circadian (LL_LDHC) gene sets were obtained from the Diurnal FTP database [42] as follows: for each set every gene that is not assigned the non-rhythmic (“n.r”) notation in the database was tagged as rhythmic, irrespective of when the peak expression is observed. The translated Arabidopsis gene ids were then overlapped with these paired diurnal and circadian gene sets and the significance of the size of overlap was tested using a hypergeometric distribution.

### Motif enrichment analysis

Promoter sets for each group of Induced, Repressed and Static groups were retrieved from the ITAG v3.2 genome assembly. The promoter was defined as the 1000 bases upstream of the transcription start site. For 424 genes (~14%), this definition overlapped with the coding region of another gene. The motif enrichment identification tool HOMER v4.10 [74] was used to identify enriched known TF binding sites in each set of promoters with the entire promoter set from ITAG v3.2 used as the background set.

### Gene ontology analysis, KEGG pathway enrichment analysis

The gene ontology (GO) enrichment (FDR < 0.05) analysis was conducted using goatools [75] with the gene-to-go associations derived from ITAG v3.2 (tomato genome consortium, Nature 2012). All genes from the tomato genome that have any GO association serve as the background for the hypergeometric significance test. The significantly enriched GO terms were further summarized using Revigo [76] (allowed similarity = 0.5). Pathway analysis was carried out using KOBAS 3.0 [77] with all tomato genes assigned to any pathway serving as the background set for significance test. The enriched pathways (adjusted p-value <0.05) were retained for all categories. GO term and KEGG pathway enrichment visualization as a heatmap (Fig. S17) was generated using pheatmap R package.

### Quantitative real-time PCR analysis

Real-time PCR was performed on RNA obtained for three biological replicates (four plants pooled per replicate). Total RNA was extracted using the RNeasy-plant-mini-kit (Qiagen, USA) and the purified total RNA was quantified using the Nanodrop ND 1000 Spectrophotometer (Thermo Fisher, USA). Genomic DNA was eliminated using the TURBO DNA-free kit (Fisher, USA) according to the manufacturer’s instructions. Each sample was further diluted to 50 ng/ml and 100ngs was utilized per reaction. Primer design was carried out using primer3 (v.0.4.0). EF1α (Solyc06g005060.2.1) was utilized as a reference gene for data normalization. Verso SYBR green one-step ROX mix Kit (Thermo Scientific, USA) was employed to perform RT-PCR according to the manufacturer’s instructions. In brief, PCR reactions were carried out in a total volume of 12.5 ml containing 2 ml of RNA, 1X SYBR green buffer and 400 nmol of each primer. The amplification reactions were carried out in a QuantStudio Flex6 thermal cycler (Thermo Scientific, USA) under the following conditions: 15 min at 50°C, 15 min at 95°C, 40 cycles of 95°C for 15 sec, 57°C for 30 sec, 72°C for 30 sec. After 40 cycles, the amplification products were heated to 95°C for 30 sec, to determine their melting curves and confirm their specificity. All amplification reactions were done with three biological replicates, and controls were included in every 96-well plate. The change in expression for the gene of interest was calculated relative to the expression of EF1α gene (i.e. reference gene), and ANOVA followed by a TukeyHSD test was used to test the significance of observed differences. The primers used for qRT-PCR analysis in this study are listed in Table S1.

### Time series gene expression assay via amplicon sequencing

Tomato seedlings were grown to 18 days old as described above and again split into two equal sets. One set was placed in a chamber set at 24°C and the other in a chamber set at 4°C and the 16 h/8 h (day/night) light cycle continued in both chambers. A group of 4 plants were sampled just prior to start of treatments and then again at 1,2,3,4,5,6,8,10,12,14,16,24 hours after transfer to ambient or cold conditions. RNA was extracted from each sample and converted to cDNA as described above. A multiplexed amplicon sequencing strategy was used to amplify and sequence reads from Ef1α (reference gene), CCA1, PRR7, RVE6, RVE8 and ELF3 from each sample. Each amplicon sequencing run yielded ~11 000–37 000 reads. Gene expression for each clock gene was calculated as ratio of reads from the gene to reads from the reference gene EF1α. A cubic spline model was fit for this relative gene expression trajectory and statistical test for significance of differential expression between 24°C and 4°C was calculated using the empirical bayes test implemented in the limma package in R as described previously [78].

### Measuring photosynthetic parameters

Tomato seedlings were grown as described above for transcriptome profiling. The MultispeQ (v2.0) instrument was then used to measure the photosynthetic parameters (i.e. ϕPSII, F_v_/F_m_, etc.) for the control (24°C) and cold treated (4°C) seedlings. The first true leaf from eight seedlings was measured at ZT2.5 (before treatment). The plants were incubated in ambient temperature or in the cold room for either 30, 90, or 180 minutes, and were returned to ambient temperature (24°C) for 5 minutes before the photosynthetic measurements were collected. The light intensity used during measurement was between 240–260 μmol m^−2^ s^−1^. The same first true leaf from eight seedlings was measured at any given timepoint, after treatment. The significance of changes in these parameters between before and after paired samples was tested by a Student’s *t*-test.

## Acknowledgements

We thank Prof. Rob McClung for critical discussions and comments and Nathan Deppe for technical assistance in experimental setup. This research was supported by funds from Purdue University.

## Author contributions

Conceptualization, investigation, and validation were conducted by T.A. and K.V. X.W. generated the CBF1 mutant and performed gene expression analysis. F.M. designed and executed the experiments to assay resumption of clock oscillation. Formal analysis and data curation were performed by T.A. and K.V. T.A. and K.V. wrote the original draft. X.W. and F.M. participated in revision and updates. I.I. and S.P. contributed resources and methodology for photosynthetic measures. All authors contributed to reviewing and editing. K.V. is responsible for funding acquisition and project supervision.

## Data Availability

All sequence and gene expression data were deposited in NCBI’s GEO database and can be retrieved with the accession number GSE226856.

## Conflict of interest statement

The authors report no conflict of interest.

## Supplementary Data

Supplementary data is available at *Horticulture Research* online.

## Supplementary Material

Web_Material_uhad137
