# Supplementary material for: Chilling stress drives organ-specific transcriptional cascades and dampens diurnal oscillation in tomato
Source: Hortic Res. 2023 Jul 11;10(8):uhad137. doi: 10.1093/hr/uhad137 (PMC10410299; doi:10.1093/hr/uhad137)
Supplement: Web_Material_uhad137 [file web_material_uhad137.zip › SupplementaryFigures_0623.pdf]

Supplementary Figures

Chilling stress drives organ specific transcriptional cascades and dampens diurnal oscillation in Tomato

Tina Agarwal, Xiaojin Wang, Frederick Mildenhall, Iskander M Ibrahim, Sujith Puthiyaveetil, Kranthi Varala

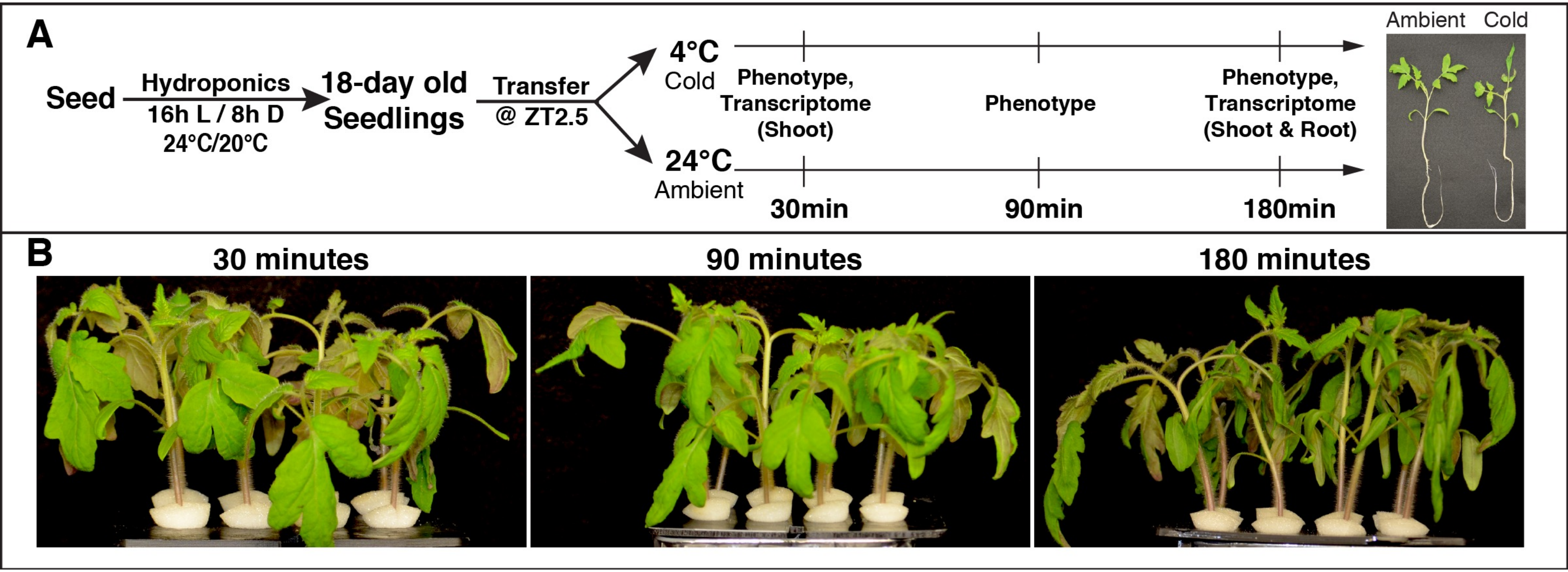

Supplementary Figure 1. Hydroponic growth allows instantaneous change of growth temperature and sampling of roots. In this study, plants growing hydroponically, in ambient conditions for 18 days, were rapidly transferred to pre-chilled containers in a cold room (4°C). Transfer was performed 2.5 hours after start of light cycle (ZT). Plants were sampled in triplicates at 30 minutes and 3 hours (180 minutes) for transcriptome profiling. Additionally, plant wilting phenotype was measured via photographs at 30, 90 and 180 minutes.

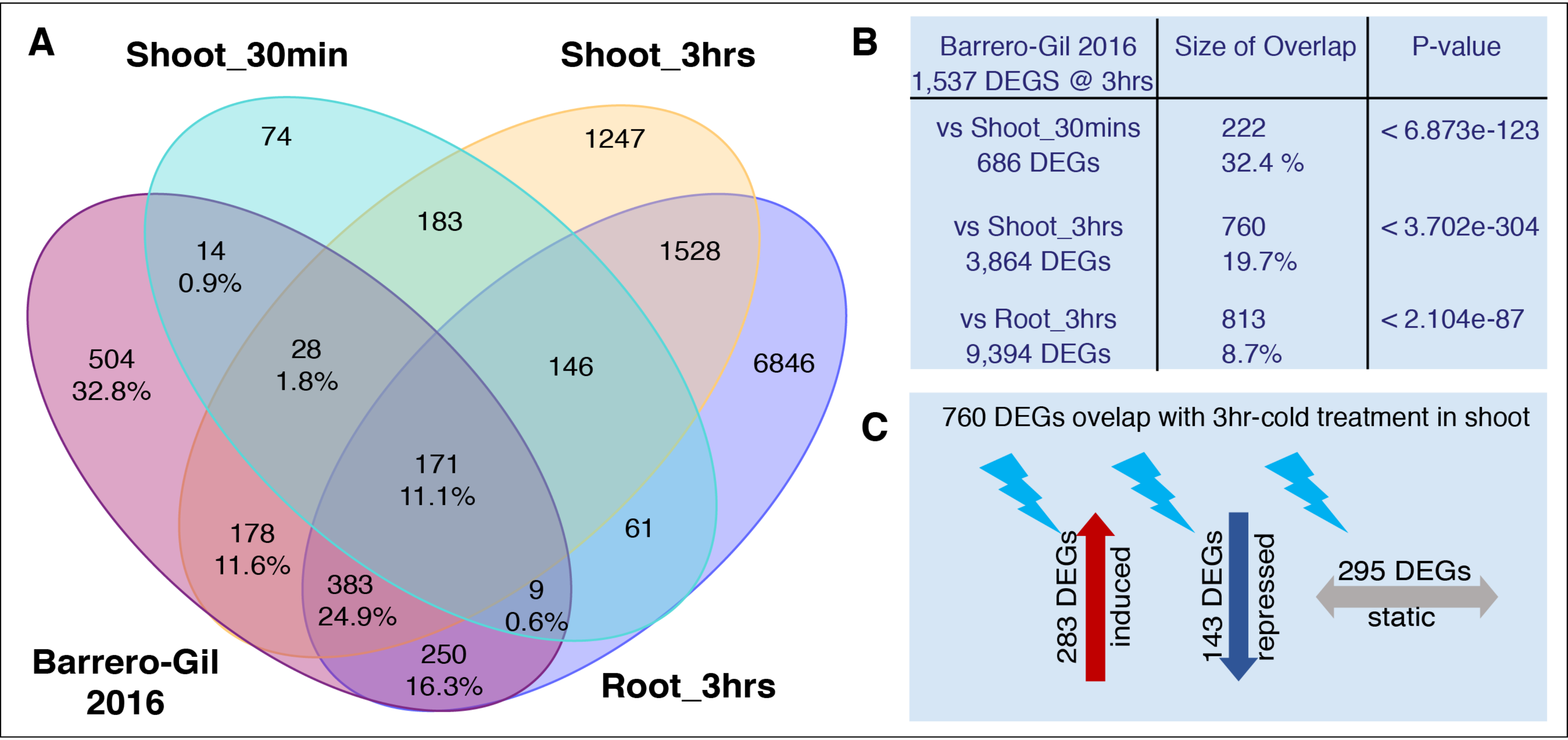

Supplementary Figure 2. Significant overlap with prior Tomato cold transcriptome study. A. Our experimental results of cold responsive genes overlap largely and significantly with a previously reported study on cold stress in tomato seedlings (Barrero-Gil et. Al., 2016). The Barrero-Gil study reported classes of cold responsive genes at 3, 6, 12 and 24 hrs after the onset of cold stress. **B.** Our study includes an earlier time point of 30 minutes as well as the crucial resolution of true cold-responsive versus cryostatic genes. The DEGs at 30 minutes (Shoots) and 3 hours (Shoots and Roots) show highly significant overlaps (hypergeometric test for significance of overlap between sets) with the 3 hr gene set in the Barrero-Gil study. **C.** Of the 760 genes that respond to cold at 3 hours in shoots in both studies, approx. 300 genes (~40%) are cold-static.

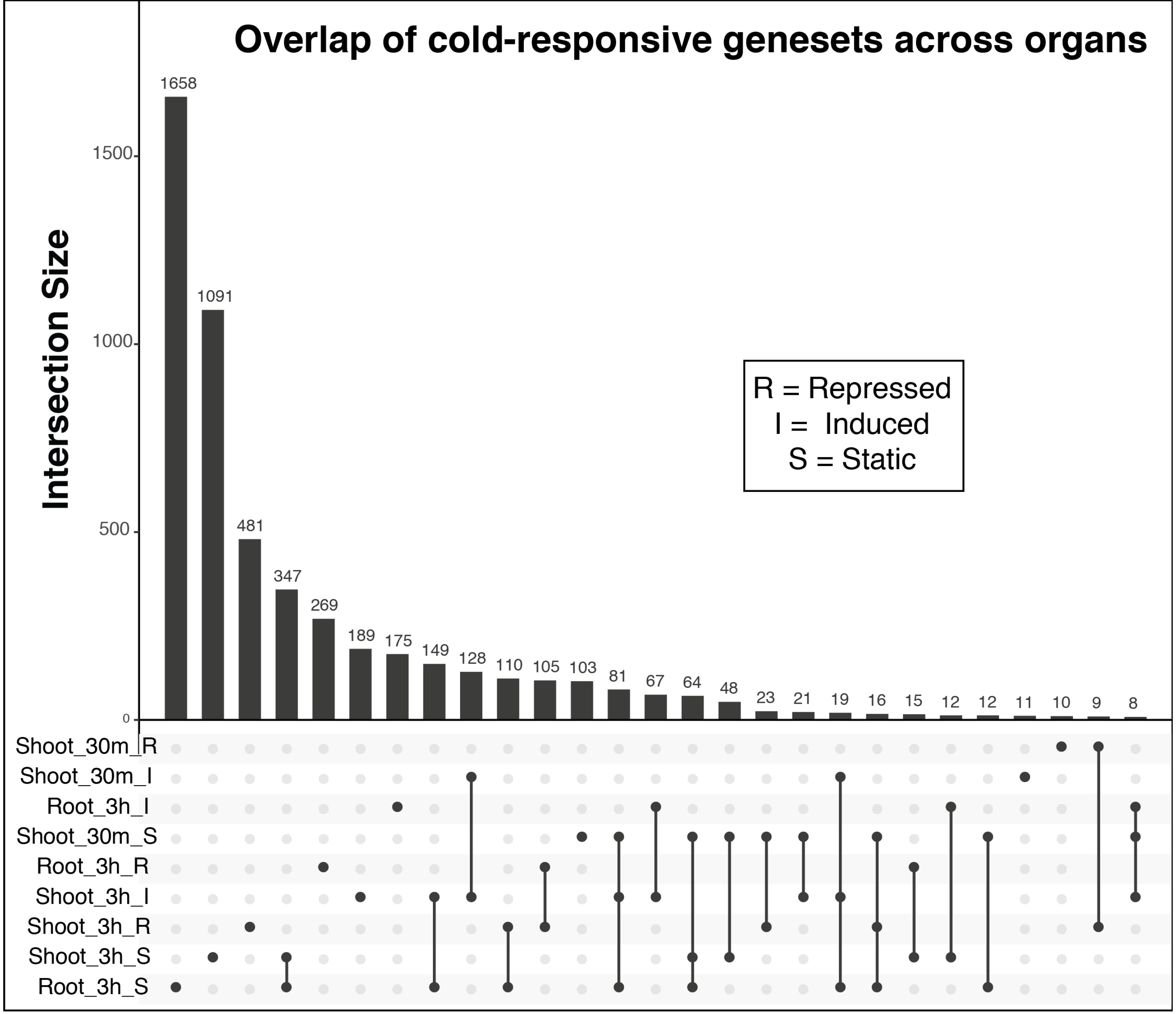

Supplementary Figure 3. Cold responsive DEGs are partially shared across organs and time points. The shoot and root cold response shares many genes across the induced, repressed and static groups. In the shoots, a small subset of genes respond early, while the later time point shows a substantially larger cold transcriptional response.

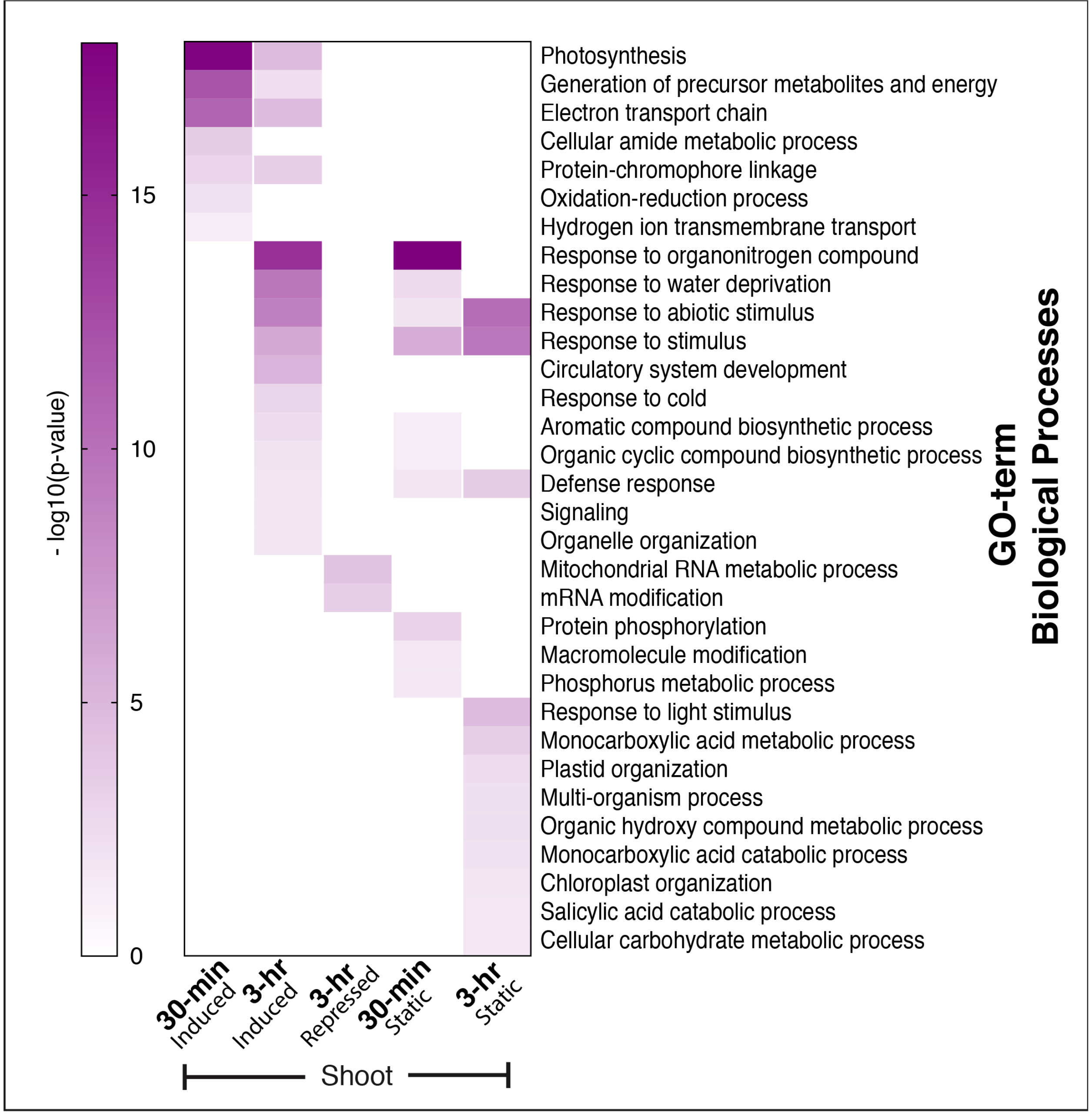

Supplementary Figure 4. ***Cold stress induces changes in multiple biological processes as determined by GO term over-representation in the DEGs in the 30 minute and 3 hour shoot samples (see Methods).*** *Genes involved in the Photosynthesis machinery respond to the cold treatment early and strongly. The stress response pathways respond within 3 hours of cold treatment. The circadian disruption caused by cold treatment has a much broader effect on plant metabolism and its regulation.*

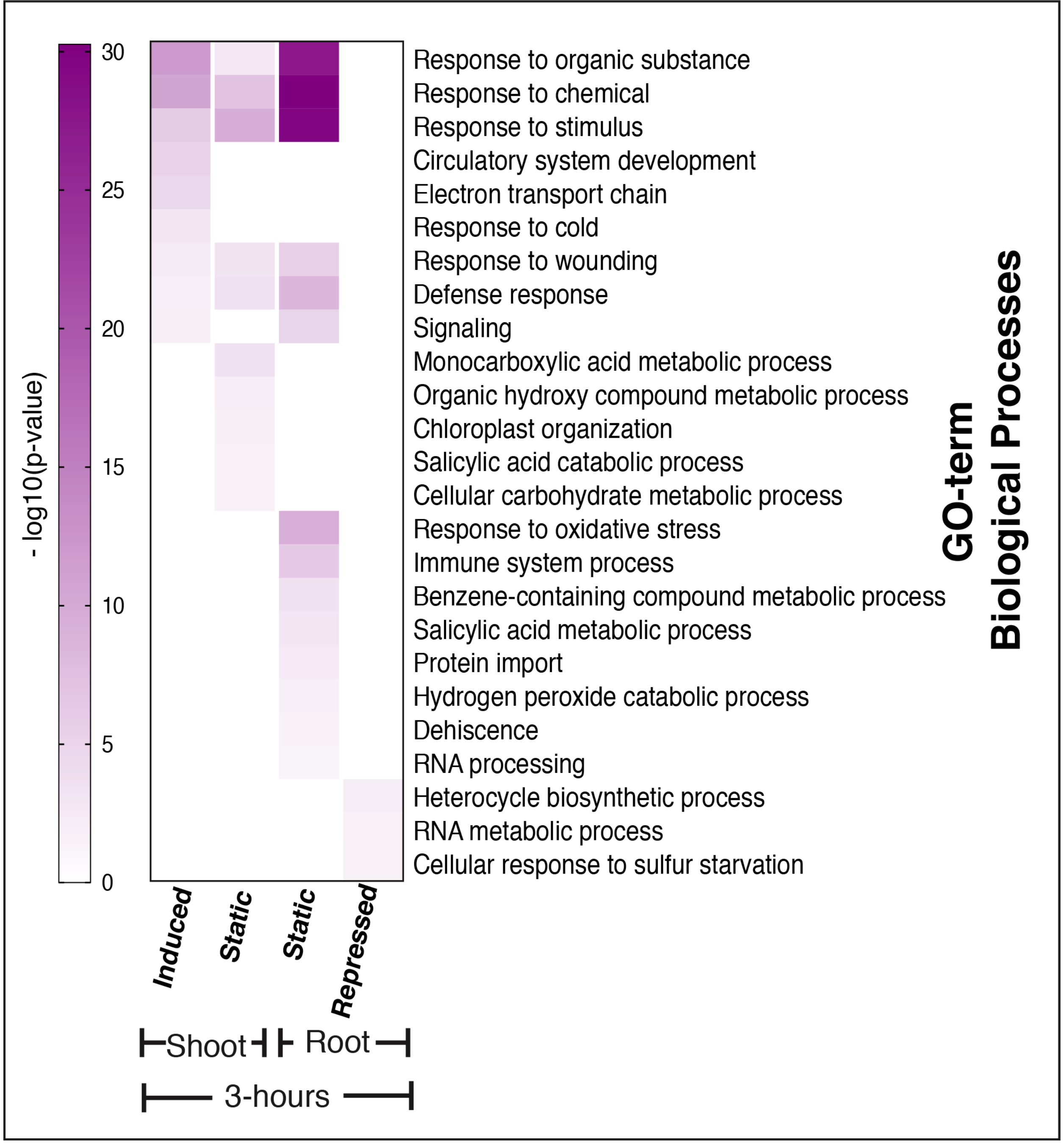

Supplementary Figure 5. ***Stress response processes are induced in the shoots and roots within 3 hours of cold treatment (see Methods)***.

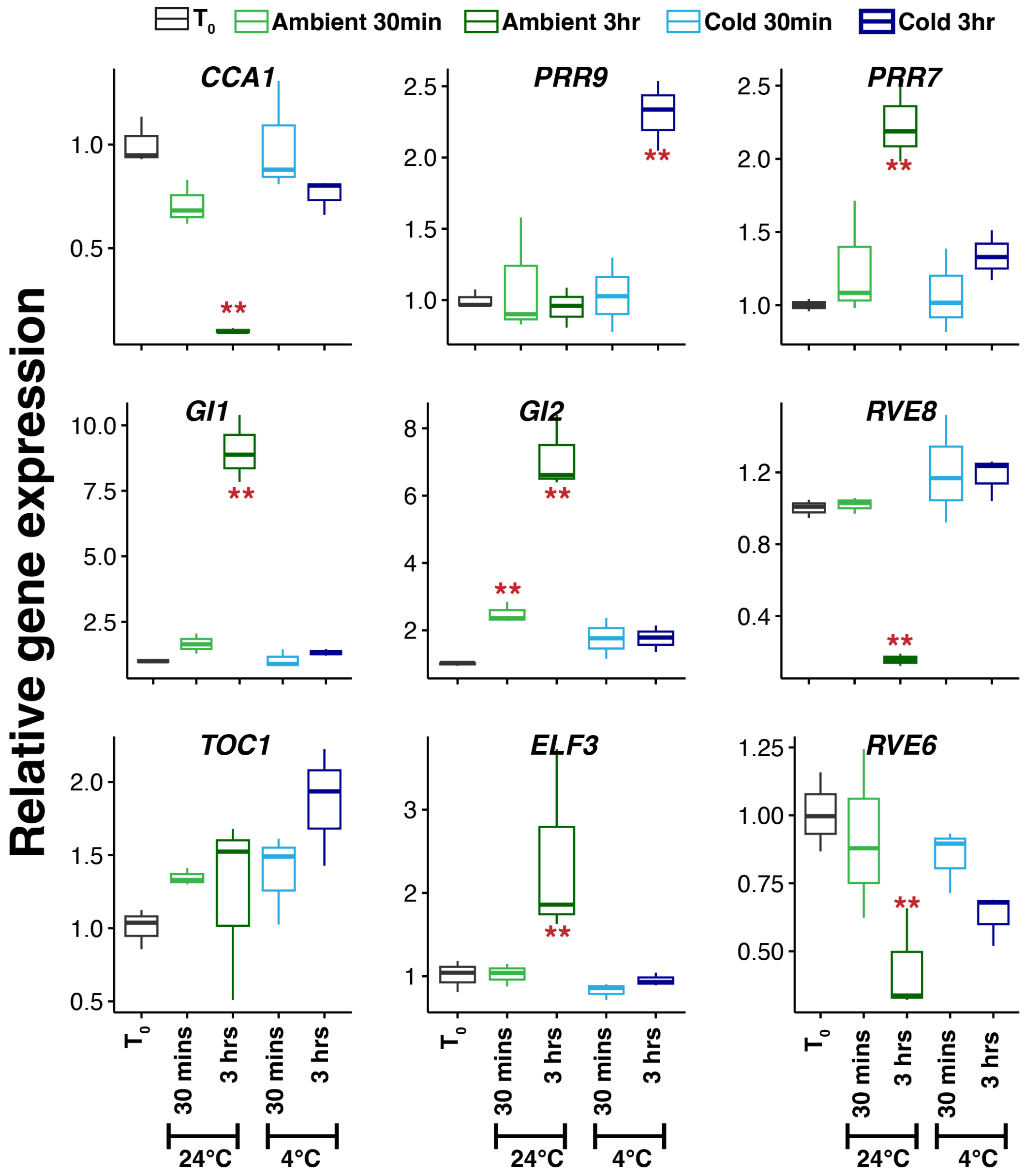

Supplementary Figure 6. Multiple core clock genes' expression is altered in chilling conditions. qPCR assays from shoot tissue using EF1a as the reference gene show altered expression patterns between plants at 24°C and 4°C. The expression level of CCA1, PRR7, GI1, GI2, RVE8, ELF3 and RVE6 was significantly different (One-Way ANOVA; Tukey HSD between each sample vs. T_0, $p<0.01$, shown as **) within 3 hours under ambient conditions. However, for all these genes, in chilling conditions there is no significant change in expression for 3 hours under chilling conditions.

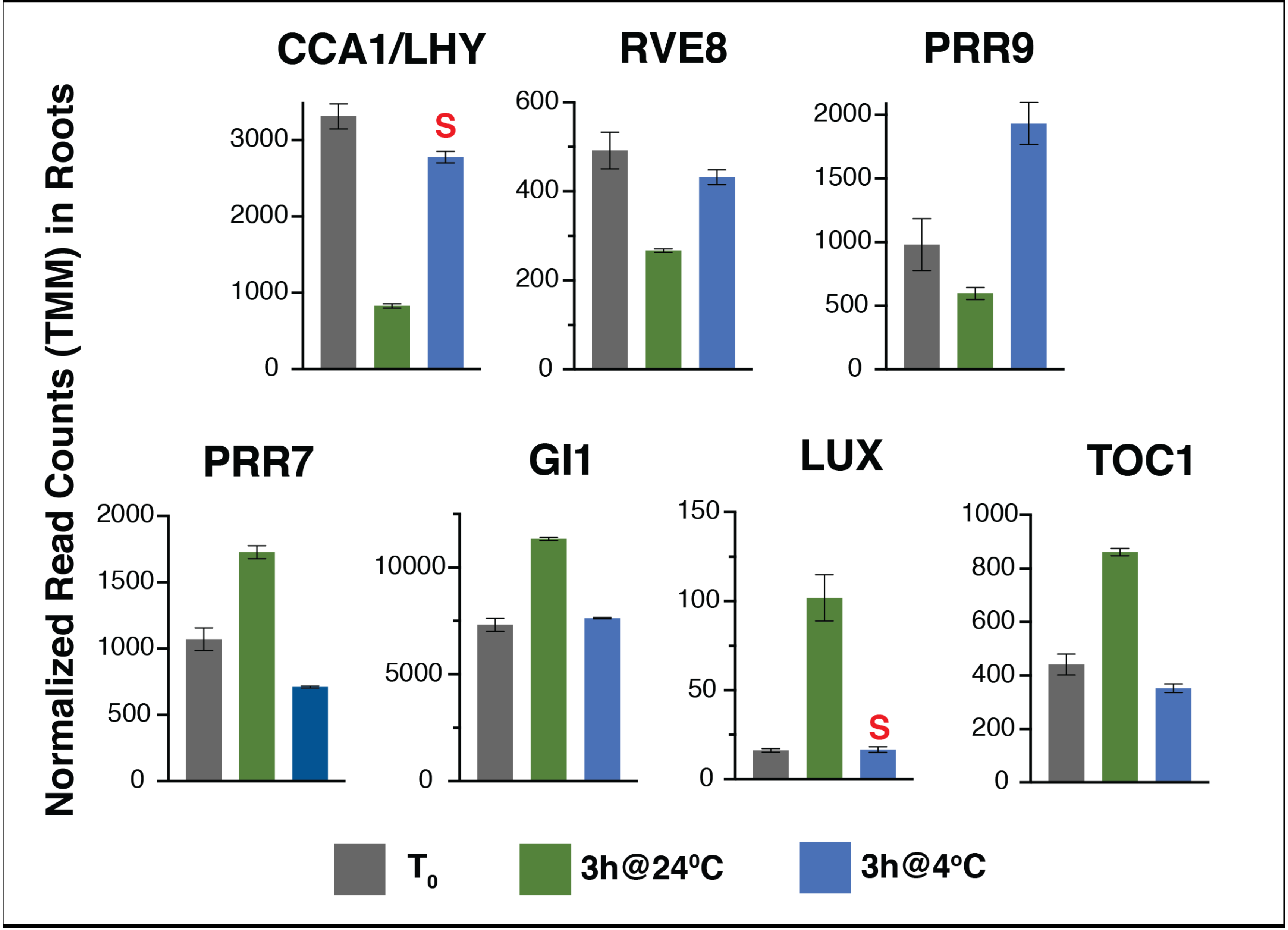

Supplementary Figure 7. Core clock components are disrupted by cold treatment in the roots as well. The rhythmic expression changes of CCA1/LHY RVE8, GI1, LUX and TOC1 are disrupted in the roots. Further, PRR9 is induced in the cold treated samples, while PRR7 is slightly repressed (Table S5). Clock genes that fall into the cryostatic category are indicated by "S".

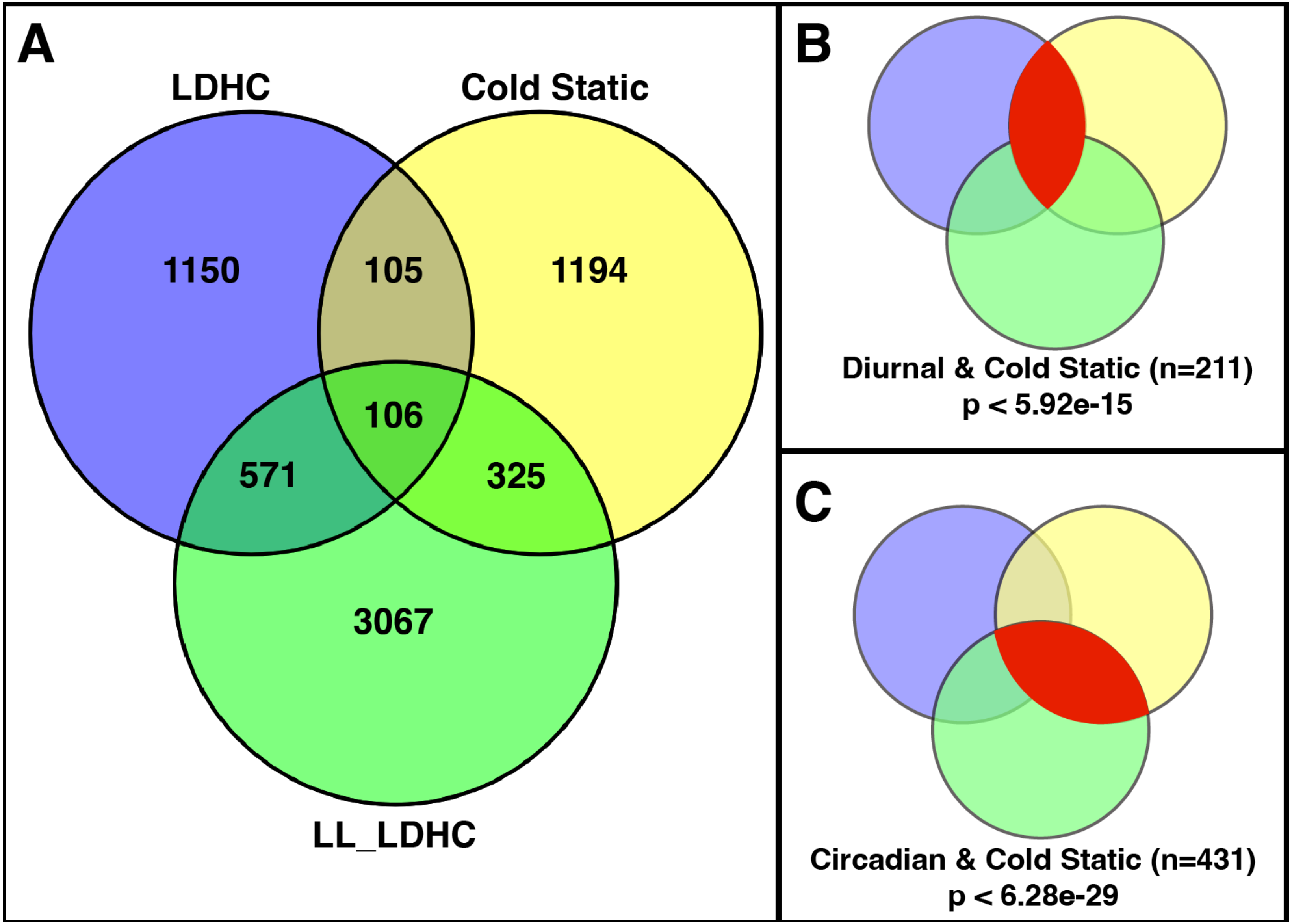

Supplementary Figure 8. Cold static gene set shows highly significant overlaps with both diurnal and circadian regulated gene sets. Tomato genes were mapped to their best Arabidopsis orthologs using the OrthoFinder algorithm (Emms and Kelly 2015). The converted gene lists were overlapped with the previously reported circadian and diurnal sets (Mockler et al., 2007). **A.** The shoot-3 hour cold-static gene set shows large overlaps with the diurnal (18h light, 8h dark) and circadian (continuous light) regulated gene sets. Overall, 62% of the cold static genes are under either diurnal and/or circadian regulation. **B.** A total of 211 cold-static genes are under dirurnal regulation which constitutes a highly significant overlap ($p < 5.9e-15$), while 431 genes ($p < 6.2e-29$) are under circadian regulation **(C)**.

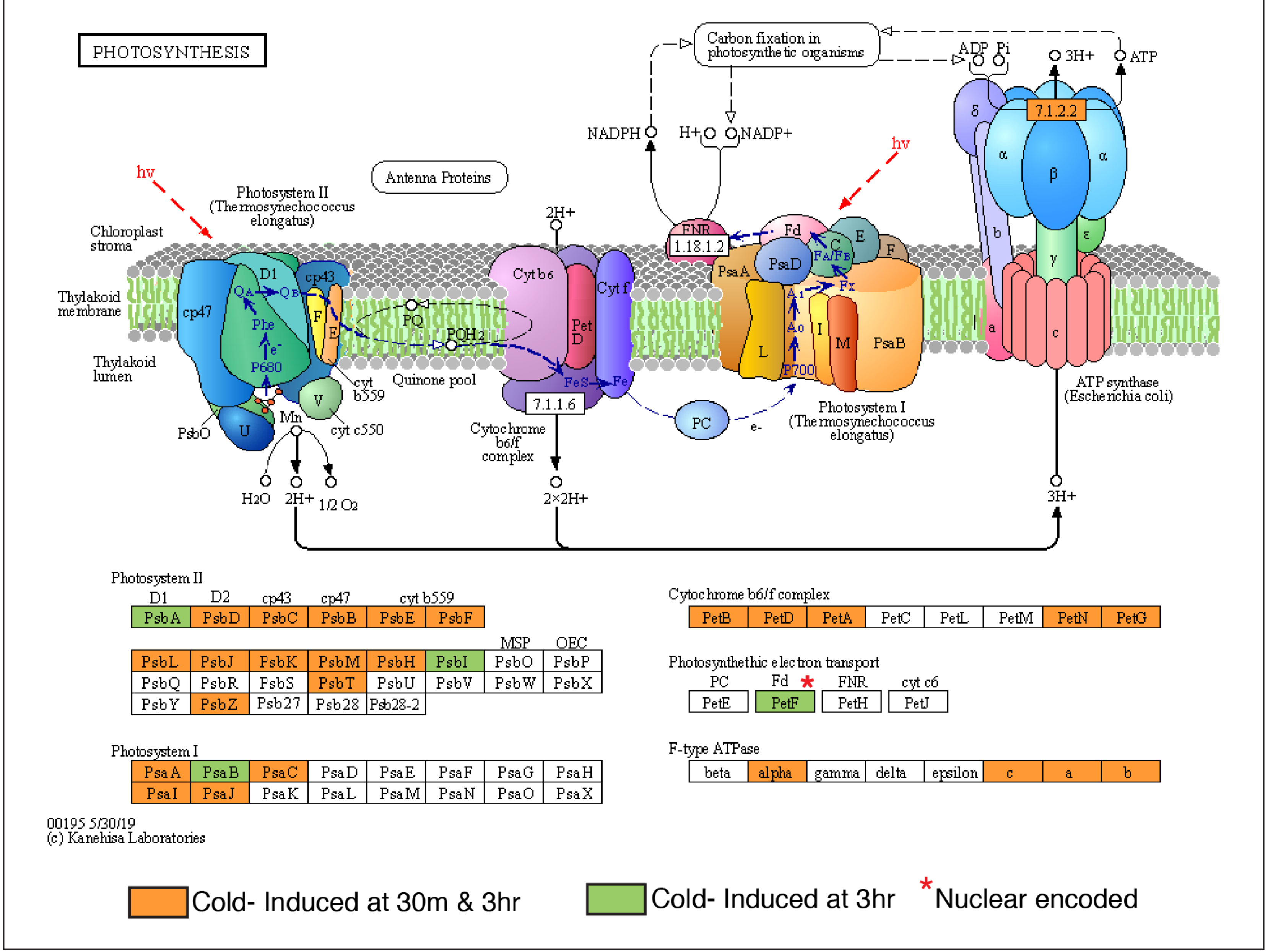

Supplementary Figure 9. Photosynthesis pathway genes induced early by cold stress. Numerous genes encoding components of PSI, PSII, Cytb$_6$f complex and F-type ATPases are significantly induced by cold treatment, within 30 minutes. With the exception of the gene coding for Fd in the electron transport chain, all the induced genes shown here are plastid encoded. Therefore, it is likely that the cold stress response includes a plastid signaling component.

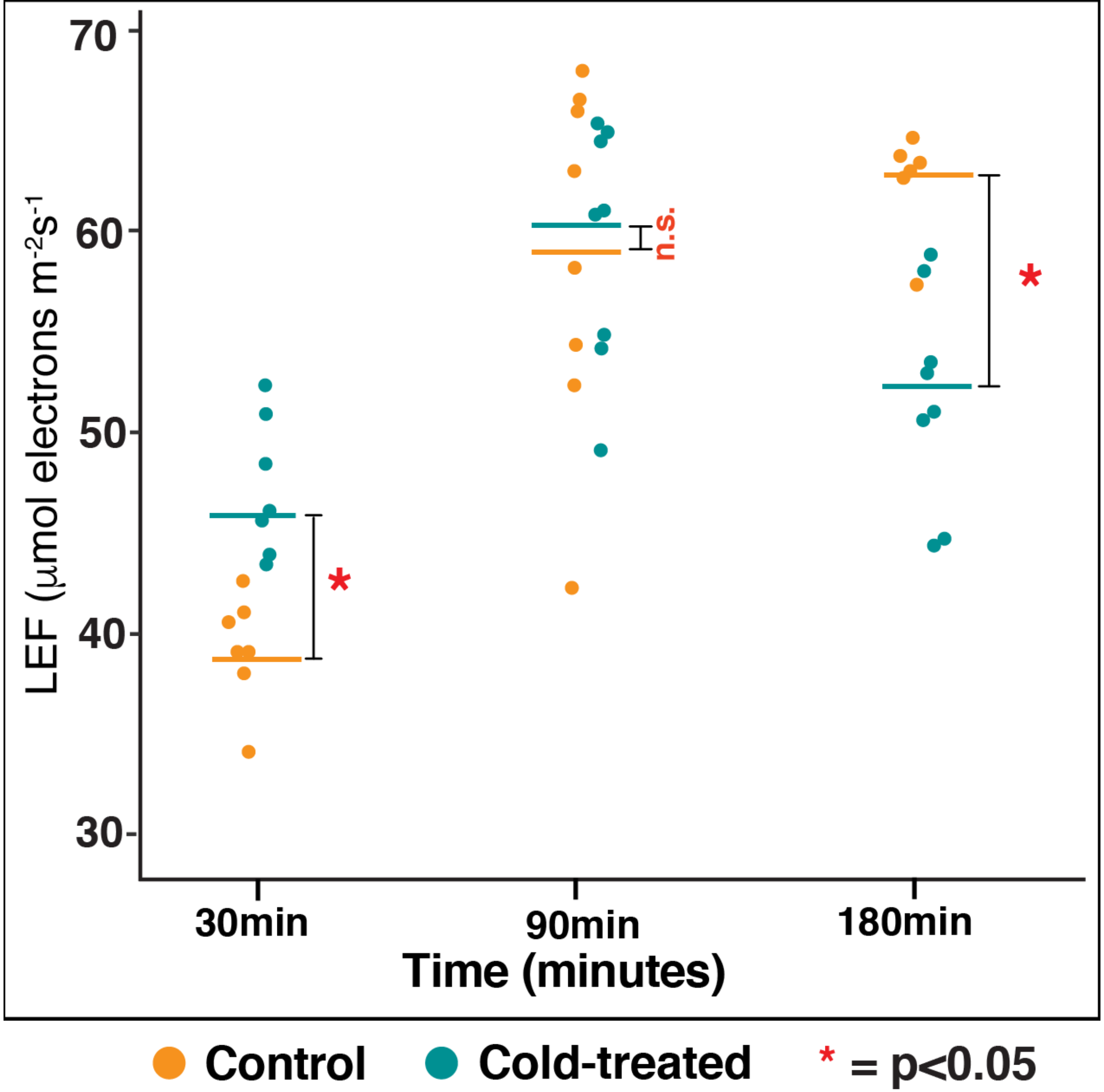

Supplementary Figure 10. Linear electron flow measurements show a strong agreement with FPSII. The LEF is a measure of the electron transport rate through the photochemical reactions and is linked to the efficiency of ϕPSII. At each of the time points measured, the pattern of change in LEF between the cold treated and control plants matched the pattern observed in ϕPSII measurements. P-values signify student's t-test for difference in means between control and cold-treated samples.

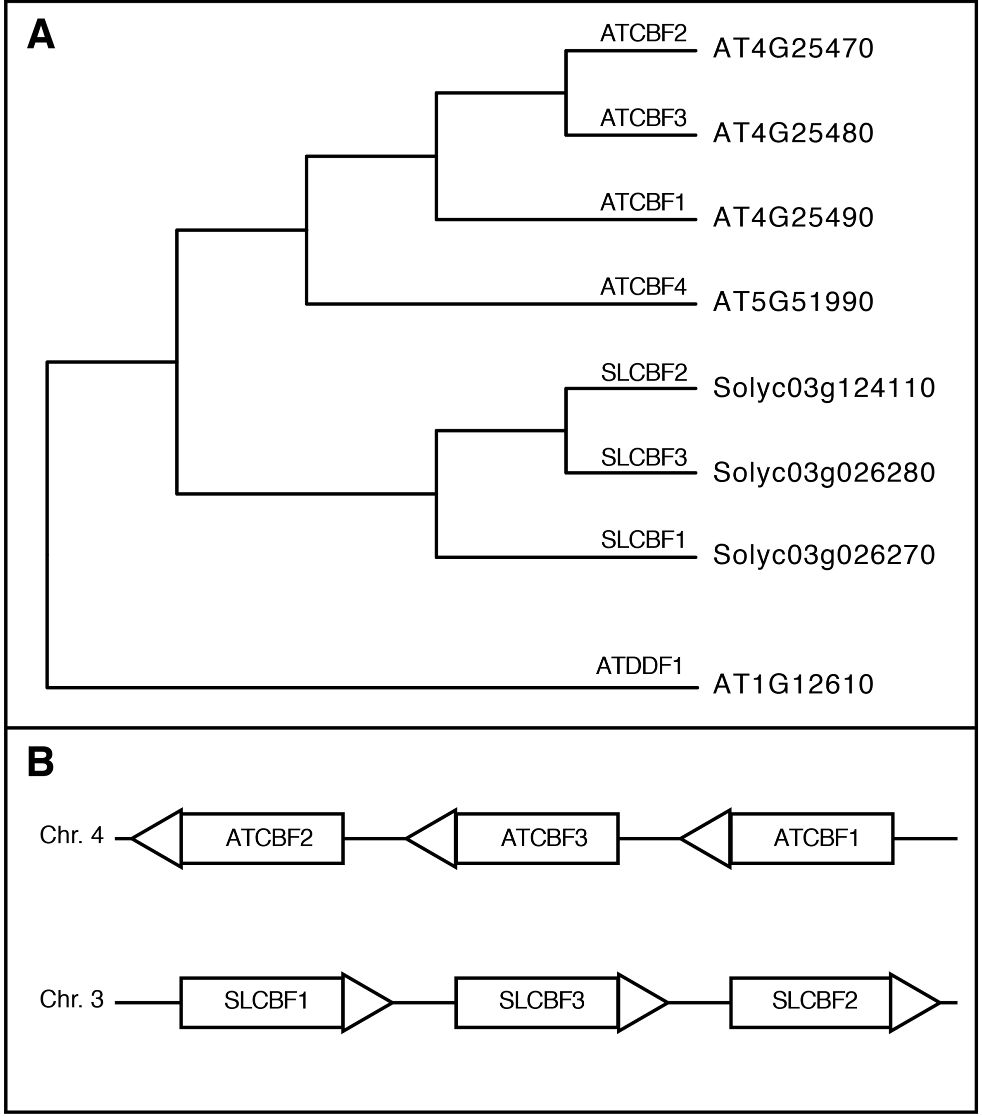

Supplementary Figure 11. Nomenclature of CBF genes in Tomato. A. Phylogenetic tree reconstruction, using maximum likelihood (ML), suggests that Solyc03g124110 and Solyc03g026280 are more closely related to each other than either to Solyc03g026270. This relationship is paralleled in Arabidopsis, wherein CBF2 and CBF3 are closer to each other than either to CBF1. ML tree reconstruction was performed using DDF1, a close ortholog of the CBFs in Arabidopsis. B. The three CBF genes in Arabidopsis and Tomato are syntenic in their respective genomes, with small intergenic regions. Assuming microsynteny is maintained at this locus the order of the three CBF genes in Tomato should match the order in Arabidopsis with CBF3 in the middle. Combining evidence from phylogenetic analysis and microsynteny, we labeled the tomato CBFs as Solyc03g026270 -> CBF1, Solyc03g124110 -> CBF2 and Solyc03g026280 -> CBF3. This notation is used throughout this report.

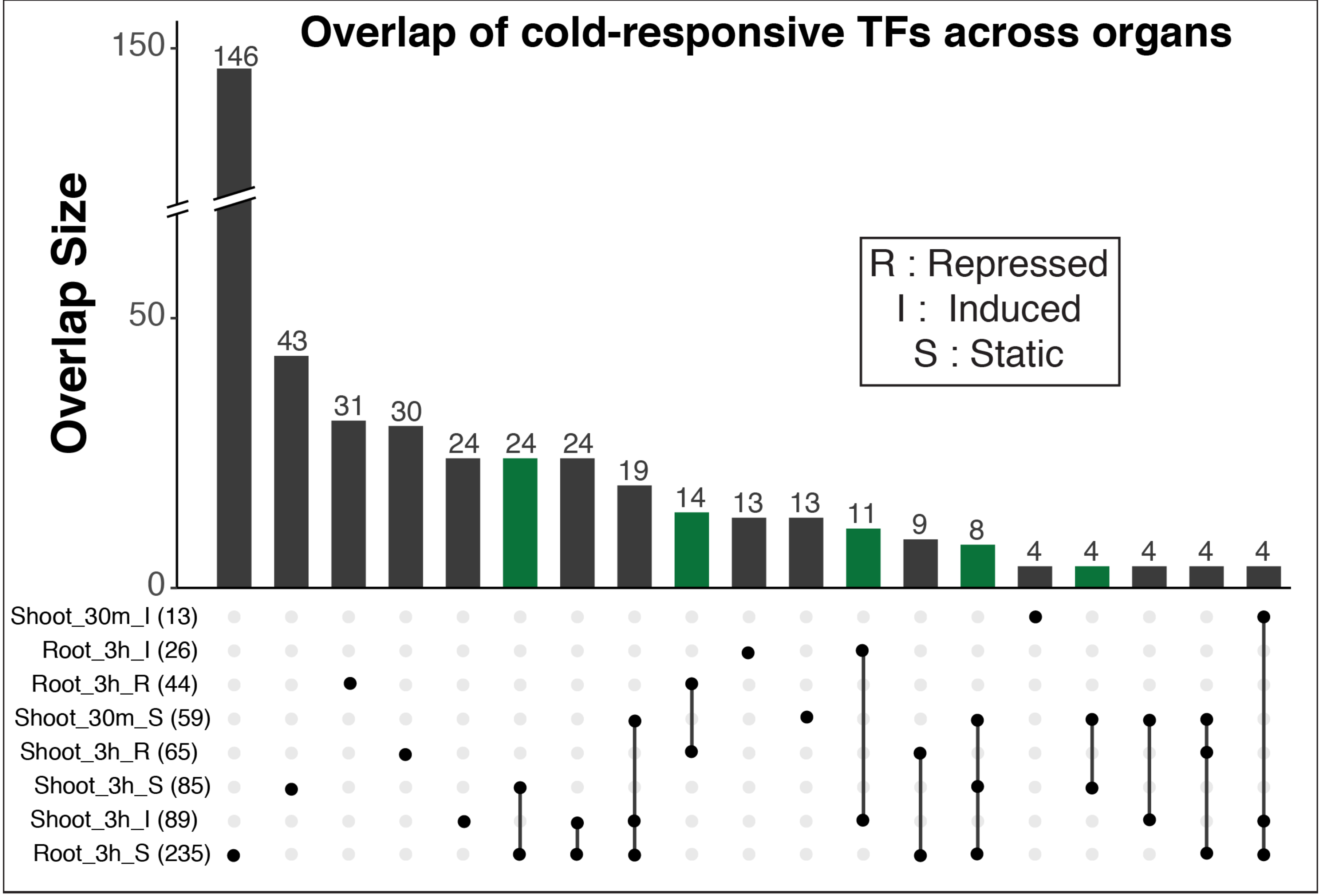

Supplementary Figure 12. Cold responsive TFs are shared across organs. Similar to the overlap of overall genesets across organs (see. Fig. S3), transcription factors (TFs) are also partially shared across organs. Many of these shared cold-responsive TFs respond in the same way across organs (highlighted in green) implying that they may have similar function across the organs. Nonetheless, the majority of TFs are either unique to the shoots or roots and potentially regulate diverse responses to chilling in the two organs.

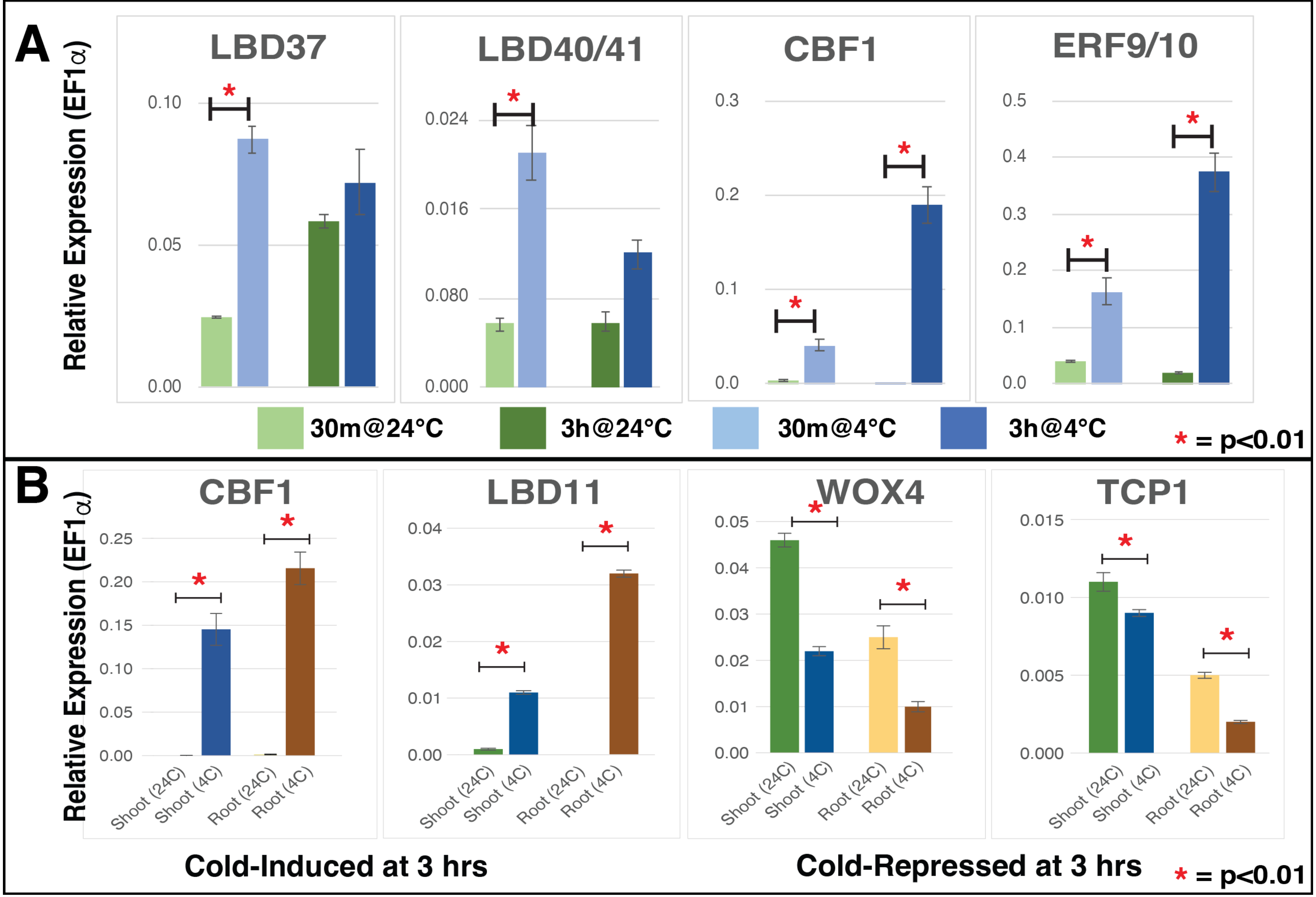

Supplementary Figure 13. Multiple Transcription Factors (TFs) show an early and robust response to chilling stress. A. Some of the early cold responsive transcription factors (TFs) in the shoots are transiently induced (e.g., LBD37, LBD40/41) while most are stably responsive to cold (e.g., CBF1, ERF9/10). **B.** Cold-responsive TFs in shoots and roots show a significant overlap, indicating a conserved cold-stress regulatory response in both organs. Shown here are select differentially expressed TFs identified by the RNA-Seq analysis that were validated via qRT-PCR using EF1α as a reference gene.

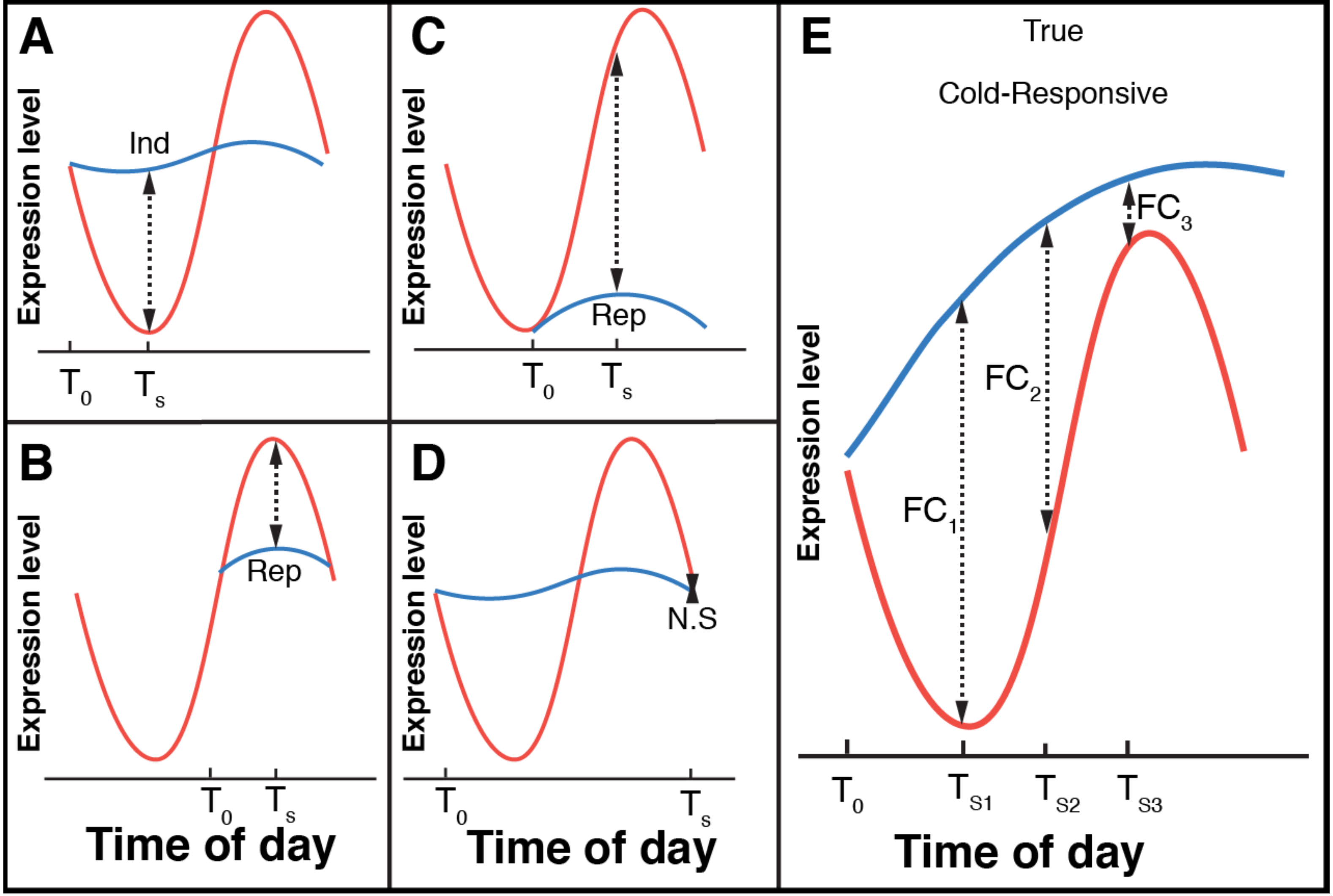

Supplementary Figure 14. Detection of a cold responsive gene that is normally under diurnal/circadian regulation is heavily influenced by the relation of the time of treatment (T_0) and the time of sampling (T_s) to the time of day. Panels A-D show how the start of chilling treatment (T0) and the time of sampling (Ts) affect the classification of the same gene as cold-induced or cold-repressed or not significant. In all cases the red line depicts the diel cycling of expression level for this sample gene and the blue line represent the static gene expression under chilling stress. **A.** Gene is detected as cold induced. **B.** Gene is classified as cold-repressed IF the amplitude of expression change is greater than the fold-change (FC) threshold applied. **C**. Gene is classified as cold-repressed with a large fold-change. **D**. Gene is classified as not differentially expressed. **E.** A true cold-responsive gene responds to the cold-stress signal and alters its expression relative to T_0. However, the observed fold-change is highly dependent on the time of sampling and a very small FC (e.g., FC_3) could prevent detection of this gene as cold-responsive. NOTE: Figure adapted from Bieniawska et. al., 2008.

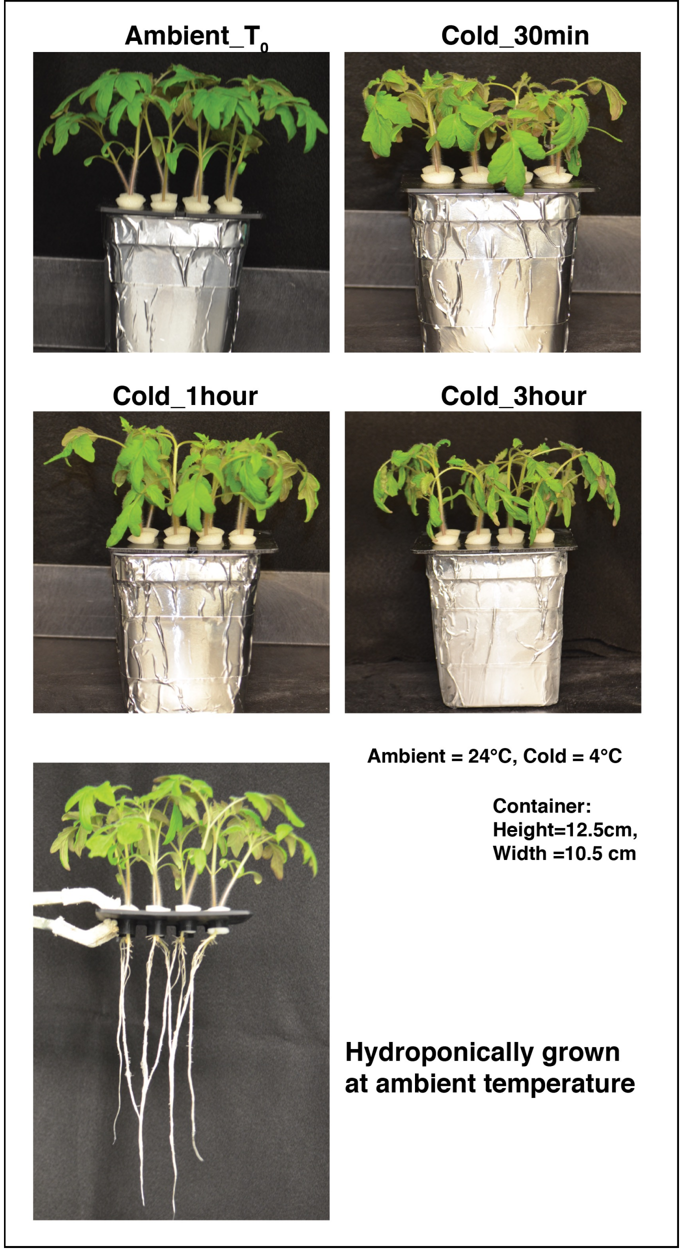

Supplementary Figure 15. Novel hydroponic setup using easily movable plant platform allows rapid transfer of plants from one growth condition to another. In this study, plants growing hydroponically in ambient conditions were rapidly transferred to pre-chilled containers of identical media stored in a cold room. This approach ensures that the shoots and roots are instantly and simultaneously exposed to a chilling temperature.

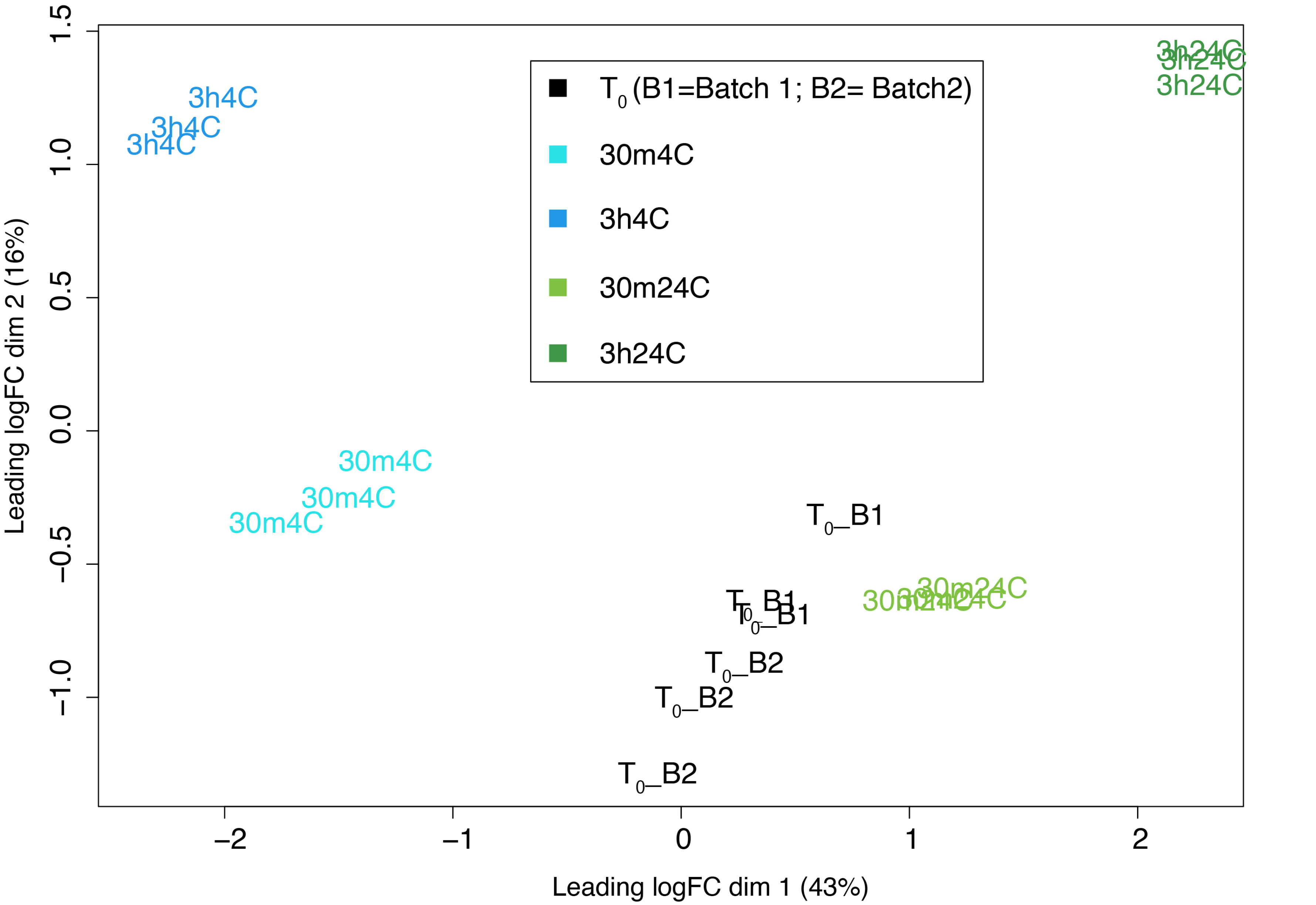

Supplementary Figure 16. Biological replicates of RNASeq from shoots cluster together. Each treatment group has three biological replicates. Shown here is the MDS plot of their global gene expression profile. The close clustering of replicates from each group and the distinct separation between groups indicates that there are large differences between the group profiles AND that the replicates are highly similar. Three T_0 samples are from Batch 1 (T_0 _B1) that included the 3 hour ambient and cold samples and another three are from batch 2 (T_0 _B2) which included the 30 minute samples. Root sample groups cluster similarly (not shown).

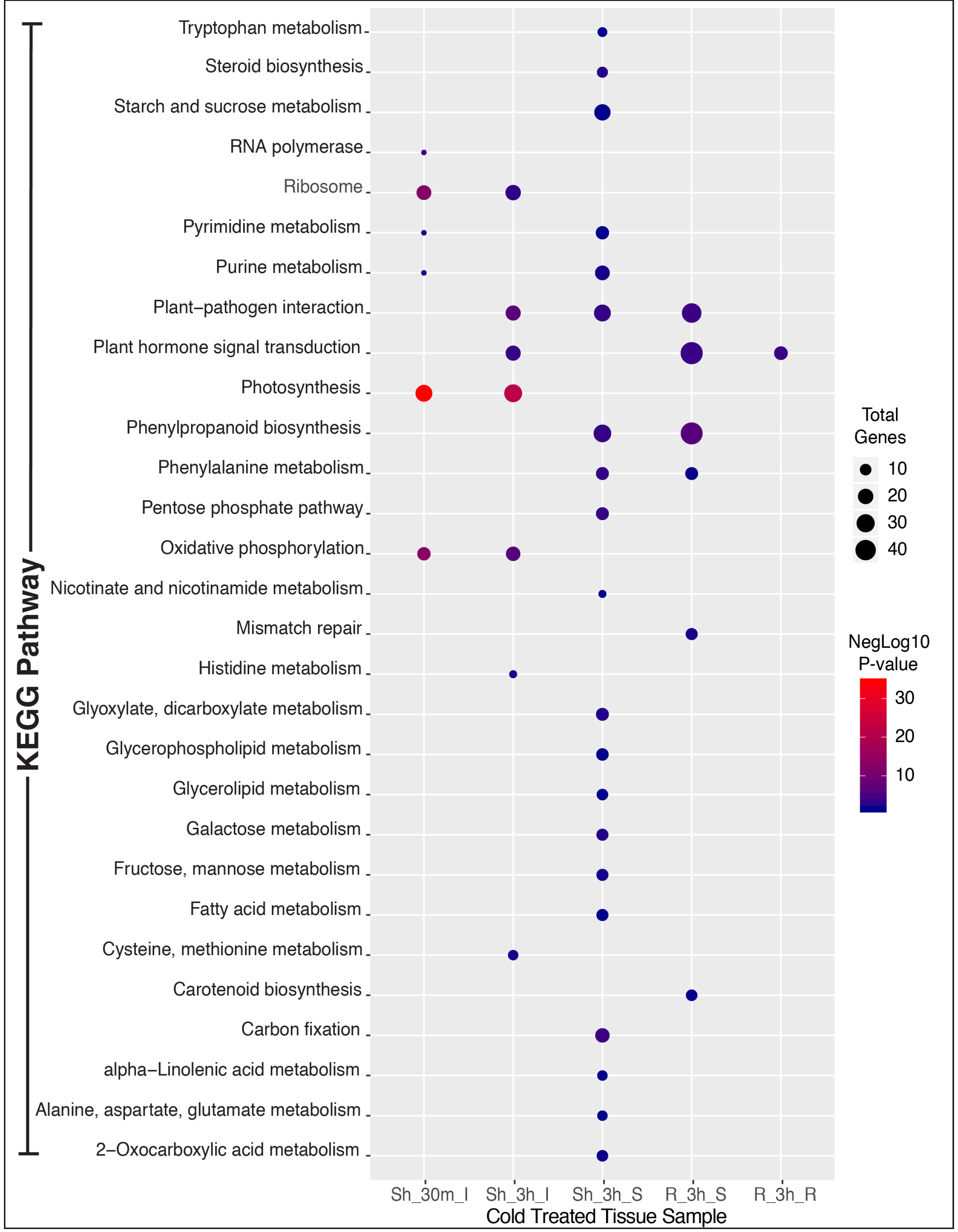

Supplementary Figure 17. Cold stress alters gene expression levels of multiple pathway components. The most significantly affected pathway is of Photosysnthesis (see Methods), where most of the genes involved in the photocenters are induced by cold. The cold-static geneset includes many primary metabolism pathways. Therefore, cold treatment is anticipated to have a major disruptive effect on cellular metabolism and hence plant physiology.

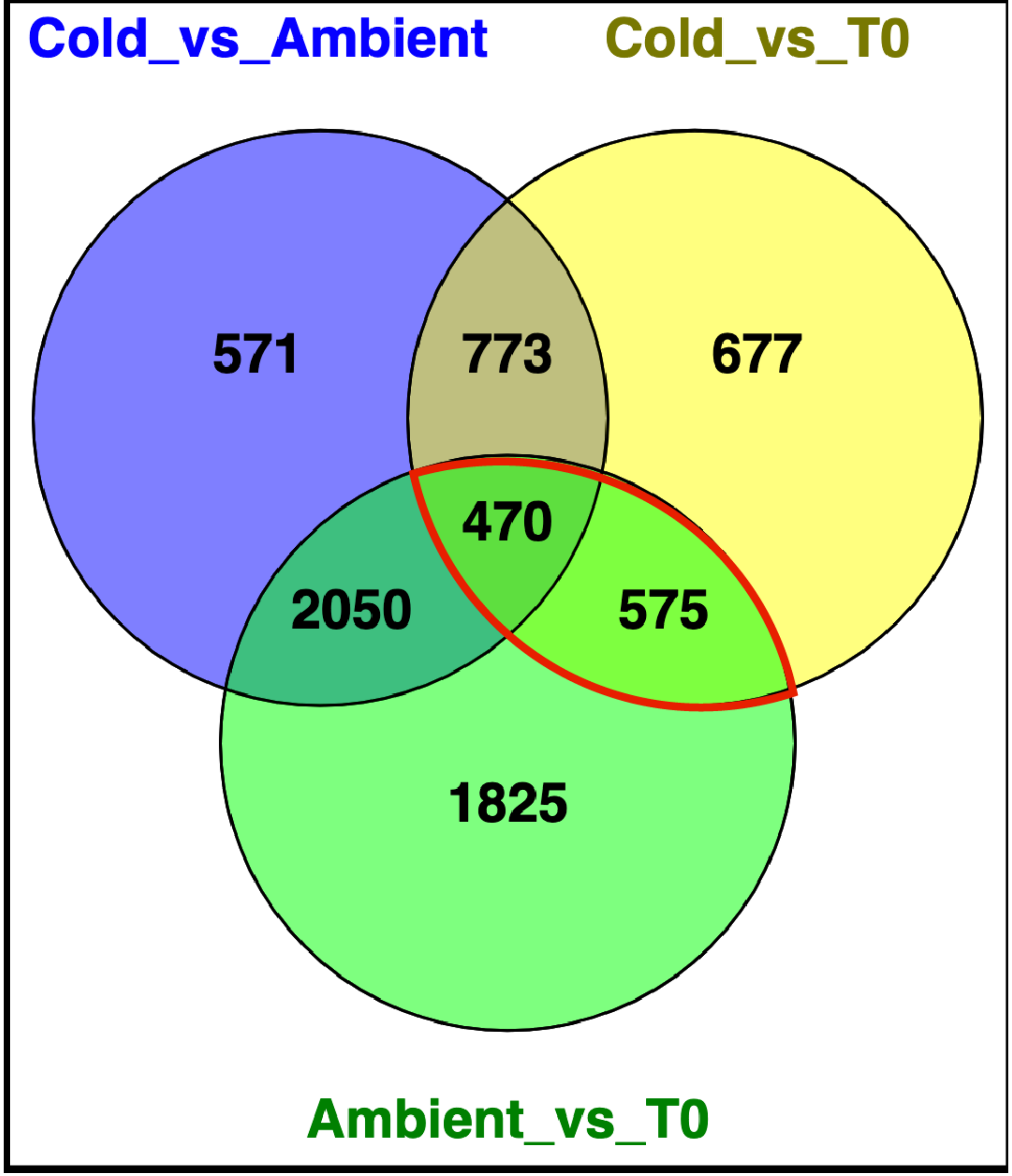

Supplementary Figure 18. Cold does not disrupt the diel oscillation of all genes. The 3-way overlap of DEGs between 1. Cold vs. Ambient, 2. Cold vs. T_0 and 3. Ambient vs. T_0 reveals 1045 genes that continue to cycle in both ambient and chilling temperatures (highlighted in red oval).
